# Burning issues: trends in newspaper coverage of wildfire events and health

**DOI:** 10.1093/pubmed/fdag010

**Published:** 2026-02-11

**Authors:** Ana Raquel Nunes

**Affiliations:** Warwick Medical School, University of Warwick, Coventry CV4 7AL, UK

**Keywords:** health, media, policy, public health, wildfire events, wildfires

## Abstract

**Background:**

The health impacts of wildfire events are a significant public health concern. Media coverage plays a central role in shaping public understanding, guiding risk perception, and influencing policy responses. However, the extent to which health-related dimensions of wildfires are integrated into media narratives remains underexplored.

**Methods:**

This study examined global newspaper coverage of wildfire-related health impacts from both quantitative and qualitative perspectives, using the Nexis Uni (LexisNexis) database and its SmartIndexing Technology. The indexed terms included ‘forest fires,’ ‘public health,’ and ‘mental health’ (i.e. Subject[Forest Fires AND Public Health AND Mental Health]), with no limits or language restrictions applied. Thematic content analysis was used to investigate how health impacts of wildfire events were framed and communicated.

**Results:**

A total of 317 newspaper articles covering wildfire events and health were published between 12 August 1993 and 24 April 2025, with media coverage increasing substantially since the late 2010s. Articles increasingly portray wildfire events as public health crisis, incorporating both physiological and psychological health risks. Narrative strategies range from technocratic framings to justice-oriented approaches highlighting Indigenous knowledge. Notably, significant disparities were observed in tone, epistemic grounding, and policy orientation between outlets. Biomedical framings were prevalent, with limited engagement with structural determinants such as climate change.

**Conclusions:**

Media coverage of wildfires has increased and shifted towards a public health framing, but remains uneven and geographically concentrated, raising questions about whether prolonged events in high-net-worth areas disproportionately drive media attention.

## Introduction

Wildfire smoke exposure has emerged as a serious public health concern,^1–7^ with growing evidence linking it to heightened risks of respiratory and cardiovascular morbidity and mortality, including aggravations of asthma, chronic obstructive disease (COPD), bronchitis, pneumonia, and cardiac.^8–14^ Particularly vulnerable populations, such as children, older adults, individuals with pre-existing health conditions, pregnant women, and socioeconomically disadvantage groups, experience disproportionate health impacts.^15–19^ Beyond acute episodes, both short- and long-term exposures to wildfire smoke are associated with systemic effects, including inflammatory and oxidative stress responses, DNA damage, and neurological and mental health outcomes.^20–23^ Of particular concern is wildfire-specific fine particulate matter (PM_2.5_: fine particulate matter with a diameter of 2.5 micrometers or less), which appears significantly more detrimental to respiratory health than PM_2.5_ from other sources, with some studies indicating up to a tenfold increase in harm.^24–30^

Despite the rising frequency and intensity of wildfire events, research on the range from acute respiratory illnesses linked to airborne particulate matter (PM_2.5_ and PM_10_: fine particulate matter with a diameter of 10 micrometers or less), to cumulative and long-term health consequences of smoke exposure for affected populations remains limited.^31^^,^^32^ Several studies emphasise the urgent need for public health interventions, improved air quality monitoring, and further research to address the growing health threats posed by wildfire events.^33–38^

Additionally, examining how the media reports on the health impacts associated with wildfire events has become increasingly urgent. Media coverage plays a critical role in shaping public understanding, informing behaviour, and influencing the prioritisation of policy responses.^39–44^ Yet, the ways in which such coverage frames health-related dimensions of wildfire events remain underexplored.

Analysing media coverage offers an opportunity to reveal the extent to which wildfire-related health risks are being communicated clearly, consistently, and in alignment with contemporary scientific and policy discourse. Examining media coverage of health impacts associated with wildfire events, is essential for several reasons reflected in current research and policy trends.^37^^,^^45^^,^^46^ Media analysis enables a systematic examination of whether reporting moves beyond describing wildfire events and emergency responses to address broader health effects, including vulnerability, adaptation, resilience, and long-term risk management.^39–44^ Through this lens, it becomes possible to evaluate whether public health narratives are being integrated meaningfully into the coverage or remain secondary to more sensational or event-driven reporting.

This study examines newspaper coverage of wildfire events and health, to understand the communication strategies used and their potential implications for health promotion, climate action, and policy development. Specifically, it investigates how newspapers construct and convey narratives linking wildfire events and health, the messaging techniques used, and the variations in these approaches across different outlets. Central to this study is the identification of dominant frames, the balance between mitigation and adaptation strategies, and the presence of temporal trends that may reflect evolving scientific understanding or policy shifts.

This analysis is grounded in the recognition that media portrayals do not merely reflect events but actively shape public perceptions and public debates. By identifying prevailing frames and narratives, the study aims to uncover gaps in the current media landscape and propose strategies to improve the communication of critical health information. Ultimately, such insight is essential for designing more effective, responsive and impactful public health campaigns, improving risk communication, and informing evidence-based policy.

The research is guided by three core questions:

- In what ways have newspapers reported on the relationship between wildfire events and health?
- How have narratives linking wildfire events and health been constructed and communicated in newspaper reporting?
- What differences can be observed across newspapers in their coverage of wildfire events and health?

By addressing these questions, this study contributes to a broader dialogue on media engagement with environmental health crises, highlighting the role of communication in mediating the intersection of public awareness, health outcomes, and policy action in the context of escalating wildfire events.

## Methods

### Data

Online and in print newspaper articles were collected from a diverse range of publications to ensure a comprehensive representation of both textual and media discourse. The articles were sourced through Nexis Uni (LexisNexis) database, using SmartIndexing Technology.^47^ The Nexis Uni (LexisNexis) database provides access to thousands of sources across different types of content, including news content. Its features include advanced filtering by publication, subject, geography, timeline, among others. The SmartIndexing Technology enables specific content retrieval by automatically indexing material based on subject relevance.^47^ The indexed terms used in the search included ‘forest fires,’ ‘public health,’ and ‘mental health’ (i.e. ‘Subject(Forest Fires AND Public Health AND Mental Health)’). This approach, using indexed terms, rather than free-text keyword searches, allowed for: (i) the exclusion of colloquial or metaphorical references to fire, such as ‘rumours spreading like wildfire’, and (ii) the inclusion of region-specific terminology such as ‘bushfire,’ thereby broadening the scope of the dataset. Duplicate articles, particularly those originating from the same newswire service and published across multiple outlets were removed using LexisNexis’s de-duplication algorithm, set to the highest similarity threshold.^48^ The search was conducted on 9^th^ May 2025, and the resulting articles form the basis for the subsequent analysis.

### Analysis

This study uses thematic content analysis from a constructivist perspective, recognising media texts as active participants in constructing societal understandings of wildfire events and health. The analytical approach adopted is grounded in the principles of content analysis as defined by Krippendorff^49^ and further informed by established methodological frameworks within the field.^50–52^ Thematic content analysis is used here as a systematic, replicable method for examining communication content, enabling the identification, categorisation, and interpretation of patterns, themes and frames within large textual datasets.^49^ This approach is particularly suited for analysing media discourse, as it allows for both quantitative assessment (e.g. frequency counts of specific frames or terms) and qualitative interpretation (e.g. contextual understanding of narratives and meanings).

The coding process began with repeated reading of the full dataset by the lead researcher to gain familiarity and inductively identify recurring patterns, issues, and discursive strategies. Summary notes were taken during the initial readings to capture relevant topics. From these, a preliminary set of codes was generated to represent emerging themes and frames. These codes were iteratively refined. This process allowed for the integration of both manifest content (i.e. explicitly stated) and latent content (i.e. implied or contextual meanings). Both qualitative and quantitative content analysis techniques were used. The qualitative component enabled the identification of narrative structures, framing devices, and thematic emphases, supporting contextual interpretation of media discourse. The quantitative component consisted of frequency counts of identified themes and frames. This mixed analytical approach ensured both depth and breadth in interpreting how wildfire events and health are portrayed in media narratives.

The analysis was reported according to the preliminary guideline for reporting bibliometric reviews of the biomedical literature (Supplementary File 1).^53^

## Results

### Description of newspaper articles

**Figure 1:**
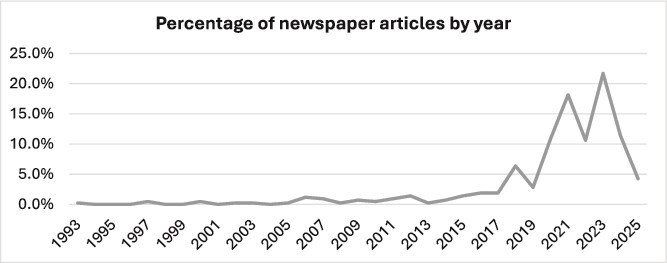
Coverage of wildfire events and health by year.

A total of 317 articles covering wildfire events and health, were published between 12 August 1993 and 24 April 2025. The data show a clear trajectory of increasing media attention to the topic or interest from 1993 to 2025 (incomplete year). Between 1993 and 2005, coverage was minimal and sporadic, with annual articles never exceeding 0.5% (n_article_ = 2 articles per year) (Fig. 1). A gradual increase began in the mid-2000s, with small increases observed in 2006 and 2007 (1.2%; n_article_ = 5 and 0.9%; n_article_ = 4, respectively), yet sustained growth did not emerge until 2016–2018, when annual coverage rose to between 1.9% (n_article_ = 8) and 6.4% (n_article_ = 27) of total coverage. An important rise began in 2020, with coverage more than tripled from the previous year, reaching 10.8%, followed by an even more substantial increase in 2021, rising to 18.2% (n_article_ = 77). The peak was observed in 2023, with 21.7% of total coverage (n_article_ = 92), the highest proportion across the entire period. Coverage remained high in 2022 and 2024 (10.6%; n_article_ = 45, and 11.3%; n_article_ = 48, respectively).

**Figure 2:**
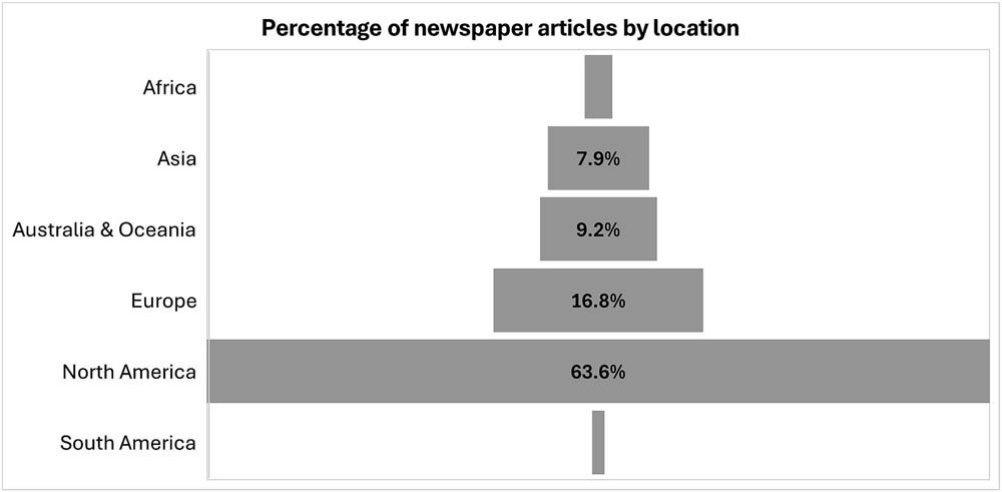
Coverage of wildfire events and health by location.

North America overwhelmingly dominates the coverage, accounting for 63.6% of the total coverage (n_article_ = 201) (Fig. 2). Europe follows with 16.8% (n_article_ = 53), while Australia & Oceania represent 9.2% (n_article_ = 29). Asia (7.9%; n = 25) and Africa (1.9%; n_article_ = 6) remain marginal in the discourse, with South America being the least represented at just 0.6% (n_article_ = 2).

**Figure 3:**
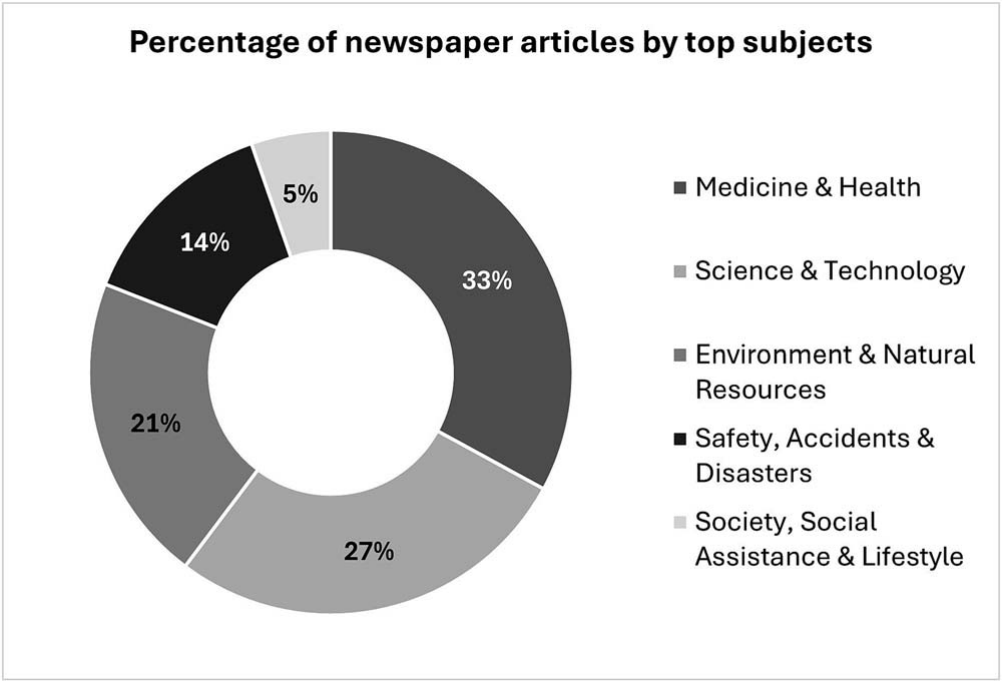
Coverage of wildfire events and health by top subjects.

As each newspaper article could be indexed under multiple subject categories, the cumulative subject counts exceed the total number of articles. The top five subjects covered include ‘Medicine & Health’ at 33.0% of the total coverage (n_subject_ = 1158). ‘Science & Technology’ follows closely at 27.3% (n_subject_ = 960), while ‘Environment & Natural Resources’ accounts for 20.7% (n_subject_ = 726), ‘Safety, Accidents & Disasters’ represents 13.7% of the coverage (n_subject_ = 483), and ‘Society, Social Assistance & Lifestyle’ is marginally represented at 5.3% (n_subject_ = 187) (Fig. 3).

**Figure 4:**
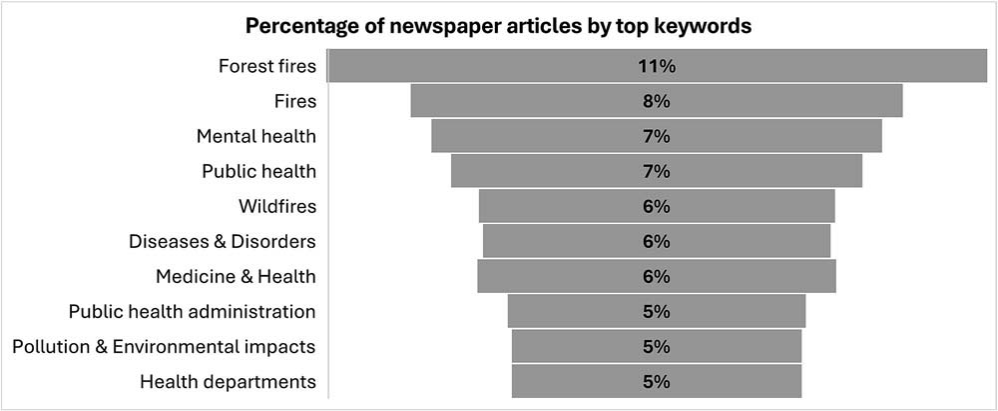
Coverage of wildfire events and health by top keywords.

The top 10 keywords in articles are led by ‘Forest fires’ (10.5%; n_keyword_ = 237) and the broader term ‘Fires’ (7.8%; n_keyword_ = 176) (Fig. 4). Major health-related terms include ‘Mental health’ (7.2%; n_keyword_ = 161), ‘Public health’ (6.5%; n_keyword_ = 147), and ‘Diseases & Disorders (5.5%; n_keyword_=124). Additionally, ‘Public health administration’ (4.7%; n_keyword_=106) and ‘Health department’ (4.6%; n_keyword_ = 103) feature notably. Environmental concerns are also represented through ‘Pollution & Environmental impacts’ (4.6%; n_keyword_ = 103).

**Figure 5:**
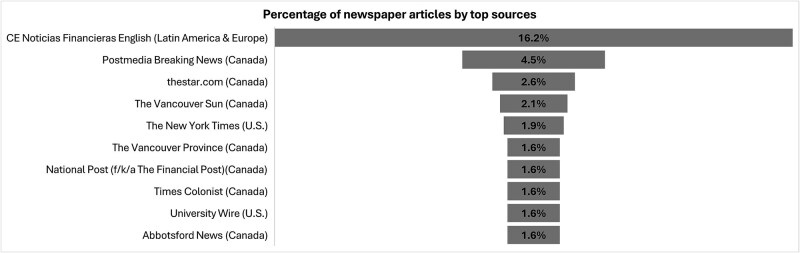
Coverage of wildfire events and health by top sources.

The distribution of top article sources is heavily concentrated, with CE Noticias Financieras English (Latin America and Europe) alone accounting for 16.2% of all articles (n_source_ = 69) (Fig. 5). Other prominent sources include Postmedia Breaking News (4.5%; n_source_ = 19) and thestar.com (2.6%; n_source_ = 11), both of which are Canadian outlets. Additional Canadian sources such as The Vancouver Sun, The Vancouver Province, National Post, Times Colonist, and Abbotsford News collectively reinforce this regional concentration. The New York Times is the only major U.S. outlet among the top 10, contributing 1.9% of articles (n_source_ = 8). The University Wire (U.S.) also appears among the top 10 sources, accounting for 1.6% of the coverage (n_source_ = 7).

### Newspaper representations of the health impacts of wildfire events

Across the dataset, wildfire events are increasingly reported as public health crisis rather than solely environmental disasters (Fig. 4). The health risks described are both physiological and psychological. Respiratory and cardiovascular conditions are frequently mentioned, particularly in relation to PM_2.5_ exposure. For example, MailOnline (UK) warns that *‘smoke billowing across state lines is filled with toxic chemicals and fine particles that can worsen asthma, lead to heart attacks and may increase the risk of developing cancer and dementia’* (Supplementary File 2, ref 1). Similarly, the Korea Herald (South Korea) reports that *‘fine particle matter (PM_2.5_) reached 537 micrograms per cubic metre’* (Supplementary File 2, ref 5), adding that these particles are ‘*significantly more toxic*’ than urban smog and capable of affecting *‘all organs in the body, including the cardiovascular system and even the brain’* (Supplementary File 2, ref 5)*.*

A strong discursive emphasis is also placed on mental health. Multiple articles highlight the emerging link between wildfire smoke and psychological distress. A Harvard TH Chan School of Public Health study cited in CE Noticias Financieras (Latin America and Europe) notes: *‘Wildfire smoke is not only a respiratory problem, but also effects mental health’* (Supplementary File 2, ref 3), associating PM_2.5_ levels with spikes in emergency department visits for depression, anxiety, and mood disorders. Medindia (India) reiterates this, emphasising that *‘women, children, young adults, Black and Hispanic individuals, and those on Medicaid’* (Supplementary File 2, ref 4) face heightened mental health risks, which exacerbate existing health inequities.

Exposure disparities are emphasised. Vulnerable populations, such as older adults, children, and structurally disadvantaged communities, are repeatedly identified. The New York Times (U.S.) article ‘Living through wildfires leaves psychological scars’ (Supplementary File 2, ref 12) illustrates how the trauma of loss, especially among the elderly, leads to *‘lasting psychological harm’* (Supplementary File 2, ref 12)*.* The cumulative picture constructed is one of multi-scalar, syndemic health impacts linked to environmental degradation and social inequality.

### Construction and communication of health and wildfire narratives in newspaper coverage

Narratives are built on a combination of scientific legitimacy, emotional resonance, and social critique. A common rhetorical method is the reframing of wildfire events as symptoms of systemic dysfunction such as policy failure, and social injustice. In an opinion piece from The Daily Titan (U.S.), wildfires are described as *‘a growing public health crisis and a symptom of our failure to address climate change’* (Supplementary File 2, ref 8)*.* The article goes on to argue for *‘primordial prevention,’* defined as targeting *‘root causes such as ecosystem degradation, unsustainable land use, in equity and climate change’* (Supplementary File 2, ref 8)*.*

Narrative elements frequently include expert authority, individual testimonies, and metaphors of crisis and transformation. The New York Times (U.S.) frames the psychological impacts of wildfire events through survivor stories, noting how a woman was *‘shellshocked*’ after watching her mother’s home burn down on television (Supplementary File 2, ref 12). The article contrasts quantitative data on health impacts with qualitative accounts of grief, displacement and eco-anxiety.

A second narrative structure foregrounds restorative justice and indigenous knowledge. The Revelstoke Times Review (Canada) reports on Ê”akisqÌ”nuk First Nation’s leadership in wildfire mitigation, where forest thinning is described not only as an ecological act but one of *‘mental wellness.’* Chief Donald Sam asserts that *‘the added benefits include residential fire safety and mental wellness’* (Supplementary File 2, ref 2), linking land stewardship to community health and intergenerational resilience. This restorative narrative critiques Western fire suppression while endorsing traditional ecological knowledge.

In contrast, tabloids such as Mirror.co.uk (UK) trivialise the crisis through sensationalism. Stories about psychics predicting wildfires lack analytical rigour, undermining the credibility of climate-health communication by reducing complex risk to entertainment spectacle (Supplementary File 2, ref 13).

### Variations in reporting practices on health and wildfire events across outlets

Differences emerge in tone, epistemological grounding and the depth of structural critique. Firstly, regarding epistemic rigour, outlets such as CE Noticias Financieras (Latin America and Europe), The New York Times (U.S.), Medindia (India), The Korea Herald (South Korea), and The Daily Titan (U.S.) rely heavily on peer-reviewed data and expert interpretation. These sources cite reputable scientific studies and institutional authorities, including JAMA Network Open (Supplementary File 2, ref 3, 4, 36), Harvard TH Chan School of Public Health (Supplementary File 2, ref 3), and UCLA (Supplementary File 2, ref 5, 13, 245, 250), thereby highlighting a commitment to evidence credibility and scientific validation. Conversely, platforms such as MailOnline (UK) and Mirror.co.uk (UK) frequently connect public health alerts in speculative projections and anecdotal narratives (Supplementary File 2, ref 13, 15).

Secondly, there is substantial geographical variation in how wildfire-related health threats are framed. Media outlets tend to emphasise region-specific challenges that shape the local experience of environmental crisis. For instance, The Korea Herald (South Korea) highlights the compounded risks posed by geographic and demographic factors, noting that certain communities in basin regions are particularly vulnerable to smoke accumulation, with elderly populations facing the greatest risk (Supplementary File 2, ref 5). In contrast, Brazilian reporting, as exemplified by CE Noticias Financieras (Latin America and Europe), frames climate disasters as both ecological and political in nature. The narrative stresses systemic governance failures, stating that recurrent fires and floods in the Midwest and North Brazil expose a chronic lack of planning and a failure to incorporate scientific expertise into public decision-making (Supplementary File 2, ref 10, 32, 164). Thirdly, the policy orientation and ideological stance of the reporting vary markedly across platforms. Some sources, such as The Daily Titan (U.S.), adopt a transformative and explicitly justice-oriented perspective, advocating for systemic change through the revitalisation of Indigenous stewardship, which is portrayed as environmental and social justice imperative (Supplementary File 2, ref 8). In contrast, more conventional outlets tend to prioritise short-term, reactive strategies, such as public health warnings and emergency response coordination. For example, The Star (Canada) emphasises measures such as issuing ‘Air Quality Health Index’ (AQHI) notices, thereby reinforcing an approach centred on mitigation rather than structural changes (Supplementary File 2, ref 89, 94). Simultaneously, several publications adopt a critical tone when addressing governmental responses, CE Noticias Financieras (Latin America and Europe) highlights political inertia and critiques austerity in mental health provision in countries such as Spain (Supplementary File 2, ref 31) and Brazil (Supplementary File 2, ref 10).

Fourthly, the narrative strategy and voice used by different outlets further shows differing approaches to reporting. Some publications adopt a colonial injustice-focused narrative, centring Indigenous leadership (Supplementary File 2, ref 2, 8, 271, 274), community resilience (Supplementary File 2, ref 8, 17, 171, 198), and trauma-informed frameworks (Supplementary File 2, ref 252, 253, 254, 259). These narratives tend to contextualise environmental hazards within broader sociohistorical patterns of marginalisation and resistance. In contrast, other sources favour a technocratic and data-driven discourse, focused on metrics such as Air Quality Index (AQI) scores (Supplementary File 2, ref 1, 47, 231) and particulate concentrations (Supplementary File 2, ref 43, 210, 243). For example, The Korea Herald (South Korea) reports on PM_2.5_ levels reaching *‘537 micrograms per cubic meter’* (Supplementary File 2, ref 5), while Medindia (India) highlights the clinical implications of exposure, noting that *‘even a modest 10 micrograms per cubic metre increase in wildfires-specific PM_2.5_’* correlates with a measurable rise in mental health-related emergency department visits (Supplementary File 2, ref 4).

## Discussion

### Main findings of the study

This study identifies a significant increase in media engagement and attention to the health dimensions of wildfire events, particularly from 2016 onwards, with a marked increase beginning in 2020. North American outlets overwhelmingly lead the coverage, suggesting a geographic imbalance in global media coverage. Thematic content analysis shows that media narratives increasingly portray wildfires as public health crisis rather than purely environmental disasters. Coverage highlights a dual burden of physical (e.g. cardiovascular and respiratory conditions) and mental health impacts, often associated with exposure to PM_2.5_. Narratives frequently cite scientific authority and expert opinion, but also include personal testimonies and cultural references, generating a hybrid framing that mixes biomedical, emotional, and justice-oriented reasonings. However, narrative coherence is not universal, it varies significantly by outlet, region, and ideological orientation.

### What is already known on this topic

Previous research has demonstrated that wildfire smoke exposure poses significant risks to human health,^8–14^^,^^54^ and disproportionately affects vulnerable populations.^15–19^ The role of the media in influencing health behaviours and public perception in general, in climate-related, as well as wildfire events crisis has also been recognised.^41^^,^^42^^,^^55–61^ However, existing literature has paid limited attention to how media narratives construct the relationship between wildfires and health, or the discursive strategies that mediate these narratives across geographies and ideological contexts.^62^

### What this study adds

This study contributes original insights into the ways media narratives frame wildfire events as syndemic threats, linking them to systemic failures in governance, social inequality, and ecological degradation. Importantly, it shows that reporting practices vary significantly across outlets, not only in tone and content, but in epistemological approach. Articles that incorporate Indigenous knowledge and restorative justice narratives offer more transformative framings than those limited to episodic reporting or technocratic descriptions. Moreover, the study highlights an undergoing biomedical and institutional bias in wildfire-health coverage, with limited integration of climate change, adaptation strategies, or structural determinants. These findings emphasise the need for more holistic, justice-based approach to media communication on climate-health intersections.

### Limitations of the study

The study draws on a single database (Nexis Uni), which although extensive, may exclude some local or non-English language sources. The analysis focuses on print and online newspapers, excluding television, radio, and social media, where wildfire-related discourse is also active and influential. Future work could extend to social media coverage and include a systematic review of the literature. The thematic coding was inductive and interpretative, which may introduce subjectivity, despite measures taken to ensure consistency and transparency. Chronological scope ends in early 2025, which limits the ability to assess coverage in response to the most recent wildfire seasons.

Whether the increased coverage reflects greater wildfire frequency and extent or heightened awareness remains unclear. While regional data on annual burned area could shed light on this, such analysis falls beyond the scope of the present study and would represent a valuable avenue for future research.

### Implications for policy and communication

Findings suggest that print and online newspapers can play a more constructive role in shaping public discourse by integrating health risks with broader environmental and social determinants.^61^^,^^63^^,^^64^ Strengthening the inclusion of marginalised voices,^17^^,^^19^^,^^63^^,^^65^ aligning coverage with scientific consensus can enhance the relevance and efficiency of health risk communication,^66–74^ and embedding policy solutions.^34^^,^^37^^,^^42^^,^^45^ There is also a clear opportunity to promote preventive and adaptive strategies in public discourse, shifting from reactive, event-based coverage to systemic, forward-looking narratives. Policymakers, public health agencies, and experts should work more proactively with media stakeholders to ensure that wildfire health risks are communicated in ways that are accurate, equitable, and actionable.

## Conclusions

This study offers timely and critical insights into the intersections of health, environment, and media. Media coverage of wildfires has risen sharply and increasingly frames them as public health crises, highlighting physical and mental health harms and unequal impacts. However, reporting is uneven, geographically concentrated, and often reactive, indicating the need for more systemic and equity-oriented communication. Future research should disentangle the extent to which prolonged wildfire events, particularly in high-net-worth areas, may disproportionately drive the volume of media coverage.

## Supplementary Material

Supplementary_File_1_BIBLIO-Checklist_fdag010

Supplementary_File_2_fdag010

## Data Availability

The data underlying this article will be shared on reasonable request to the corresponding author.

## Notes

**Ana Raquel Nunes,** Associate Professor in Health and Environment
