# Supplementary material for: Burning issues: trends in newspaper coverage of wildfire events and health
Source: J Public Health (Oxf). 2026 Feb 11;48(2):563–71. doi: 10.1093/pubmed/fdag010 (PMC13223581; doi:10.1093/pubmed/fdag010)
Supplement: Supplementary_File_2_fdag010 [file supplementary_file_2_fdag010.pdf]

Bibliography

1. Ellyn Lapointe For Dailymail.Com. (April 24, 2025 Thursday). 10million Americans told not to leave their homes TODAY as toxic chemicals sparks air quality emergency. *MailOnline.* https://advance.lexis.com/api/document?collection=news&id=urn:contentItem:6FN1-4F63-RXGM-728B-00000-00&context=1519360.

2. (April 22, 2025). First Nation spearheads 50 hectares of wildfire mitigation near Invermere. *Kimberley Daily Bulletin (Cranbrook).* https://advance.lexis.com/api/document?collection=news&id=urn:contentItem:6FMM-DRS3-RSSM-R3VY-00000-00&context=1519360.

3. (April 8, 2025 Tuesday). Why wildfire smoke affects mental health, Harvard experts say. *CE Noticias Financieras English.* https://advance.lexis.com/api/document?collection=news&id=urn:contentItem:6FHS-F423-RS3D-D030-00000-00&context=1519360.

4. (April 8, 2025 Tuesday). Wildfires Aren't Just Burning Forests-They're Igniting a Mental Health Crisis. *Medindia.* https://advance.lexis.com/api/document?collection=news&id=urn:contentItem:6FHJ-HKP3-RYKW-00WH-00000-00&context=1519360.

5. (March 28, 2025 Friday). After the fires, other threats persist . *THE KOREA HERALD.* https://advance.lexis.com/api/document?collection=news&id=urn:contentItem:6FF7-5PS3-RW5N-D2NS-00000-00&context=1519360.

6. (March 14, 2025 Friday). New list of world's most polluted countries revealed. *CE Noticias Financieras English.* https://advance.lexis.com/api/document?collection=news&id=urn:contentItem:6FBF-5MC3-RVTR-Y560-00000-00&context=1519360.

7. (February 6, 2025 Thursday). Trump's follies are already impacting Brazil. *CE Noticias Financieras English.* https://advance.lexis.com/api/document?collection=news&id=urn:contentItem:6F2S-CTF3-RS2M-B4VS-00000-00&context=1519360.

8. (February 5, 2025 Wednesday). Letter to the Editor: Recent LA fires signal a greater climate issue | Opinion | dailytitan.com. *The Daily Titan: California State University - Fullerton.* https://advance.lexis.com/api/document?collection=news&id=urn:contentItem:6F2H-D3D3-RS8M-754T-00000-00&context=1519360.

9. (February 1, 2025 Saturday). Yolanda Díaz exhibits a psychic support plan for those affected by "climate change" while no aid arrives in Valencia. *CE Noticias Financieras English.* https://advance.lexis.com/api/document?collection=news&id=urn:contentItem:6F1R-1HD3-S22D-5256-00000-00&context=1519360.

10. (January 29, 2025 Wednesday). Climate disasters and mental health: it's time to listen to science. *CE Noticias Financieras English.* https://advance.lexis.com/api/document?collection=news&id=urn:contentItem:6F12-M293-RVHY-C3T6-00000-00&context=1519360.

11. (January 20, 2025 Monday). Fires open the door to a health crisis. *CE Noticias Financieras English.* https://advance.lexis.com/api/document?collection=news&id=urn:contentItem:6DY4-W9M3-S94H-944H-00000-00&context=1519360.

12. By Emily Schmall and Katie Mogg. (January 10, 2025 Friday). Living Through Wildfires Leaves Psychological Scars. *The New York Times.* https://advance.lexis.com/api/document?collection=news&id=urn:contentItem:6DVS-VM43-RRWS-30BS-00000-00&context=1519360.

13. By, Julia Banim. (January 10, 2025 Friday). Psychic who 'predicted Notre Dame fire' made eerie 2025 weather prediction before LA flames. *mirror.co.uk.* https://advance.lexis.com/api/document?collection=news&id=urn:contentItem:6DVV-9N23-RW3W-M1R1-00000-00&context=1519360.

14. Emily Schmall and Katie MoggEmily Schmall is a reporter for the section of The Times with a focus on stories about climate and health.. (January 9, 2025 Thursday). Wildfires Can Leave Lasting Psychological Scars. *The New York Times* . https://advance.lexis.com/api/document?collection=news&id=urn:contentItem:6DVM-BS03-RXVJ-14TS-00000-00&context=1519360.

15. Matthew Phelan Senior Science Reporter For Dailymail.Com. (January 2, 2025 Thursday). Psychic who predicted Covid reveals terrifying forecasts for 2025. *MailOnline.* https://advance.lexis.com/api/document?collection=news&id=urn:contentItem:6DT8-J9J3-S603-C2BM-00000-00&context=1519360.

16. (December 20, 2024 Friday). Wildfires: a silent health threat. *CE Noticias Financieras English.* https://advance.lexis.com/api/document?collection=news&id=urn:contentItem:6DPS-7NC3-S08R-0455-00000-00&context=1519360.

17. China Daily. (November 26, 2024 Tuesday). Optimizing climate health adaptation policies. *China Daily.* https://advance.lexis.com/api/document?collection=news&id=urn:contentItem:6DH5-5GH1-F11P-X4RV-00000-00&context=1519360.

18. (November 13, 2024 Wednesday). Rare and orphan disease patients have not been regulated by law for more than 480 days.. *CE Noticias Financieras English.* https://advance.lexis.com/api/document?collection=news&id=urn:contentItem:6DDM-VPX1-DYY9-021G-00000-00&context=1519360.

19. The Editorial Board. (November 11, 2024 Monday). Feverish state: Editorial on the impact of climate change on health and India's economy. *The Telegraph (India).* https://advance.lexis.com/api/document?collection=news&id=urn:contentItem:6DD1-X481-JB5M-W27H-00000-00&context=1519360.

20. (November 7, 2024 Thursday). Climate change has doubled the number of fires and there are concerns about their growing impact on health.. *CE Noticias Financieras English.* https://advance.lexis.com/api/document?collection=news&id=urn:contentItem:6DCC-1R01-DYY9-00N9-00000-00&context=1519360.

21. (October 23, 2024 Wednesday). An expert reveals the impact of climate change on health: "It affects the balance of...". *CE Noticias Financieras English.* https://advance.lexis.com/api/document?collection=news&id=urn:contentItem:6D85-98V1-JCG7-83N8-00000-00&context=1519360.

22. Marc Kitteringham. (October 9, 2024). Arzeena Hamir - B.C. Green Party. *Comox Valley Record.* https://advance.lexis.com/api/document?collection=news&id=urn:contentItem:6D52-4FK1-F0HF-80CD-00000-00&context=1519360.

23. (October 8, 2024 Tuesday). Minsa stresses the importance of physical activity to protect mental health. *CE Noticias Financieras English.* https://advance.lexis.com/api/document?collection=news&id=urn:contentItem:6D55-PPD1-DYY9-041B-00000-00&context=1519360.

24. Marc Kitteringham. (October 4, 2024). Election Supplement - Arzeena Hamir - B.C. Green Party. *Comox Valley Record.* https://advance.lexis.com/api/document?collection=news&id=urn:contentItem:6D40-84N1-

JD2C-J3RC-00000-00&context=1519360.

25. Sayra Cristancho. (September 27, 2024). How better community engagement can improve emergency management in Canada. *Yukon News.* https://advance.lexis.com/api/document?collection=news&id=urn:contentItem:6D2M-4CP1-JD2C-J4F9-00000-00&context=1519360.

26. (September 25, 2024 Wednesday). Active fires in Quito, what is the mind of an arsonist like?. *CE Noticias Financieras English.* https://advance.lexis.com/api/document?collection=news&id=urn:contentItem:6D26-4WD1-DYY9-03NG-00000-00&context=1519360.

27. (September 10, 2024 Tuesday). Learn about the health effects of air pollution and how to protect yourself. *CE Noticias Financieras English.* https://advance.lexis.com/api/document?collection=news&id=urn:contentItem:6CY0-JMT1-JCG7-840N-00000-00&context=1519360.

28. Stephanie Cleland and Ryan W. Allen. (August 4, 2024 Sunday). Getting to the bottom of a burning question; Sunday Op-Ed; As wildfires become more frequent, how will persistent smoke exposure affect long-term health?. *The Vancouver Province (British Columbia).* https://advance.lexis.com/api/document?collection=news&id=urn:contentItem:6CMW-0CT1-DYWW-80R3-00000-00&context=1519360.

29. (August 4, 2024 Sunday). Wildfire smoke and its impact on brain health, experts say. *CE Noticias Financieras English.* https://advance.lexis.com/api/document?collection=news&id=urn:contentItem:6CN3-MBV1-JCG7-80F9-00000-00&context=1519360.

30. Hardip Johal. (August 2, 2024 Friday). Opinion: As wildfires become more frequent, how will persistent smoke exposure affect long-term health?. *Postmedia Breaking News.* https://advance.lexis.com/api/document?collection=news&id=urn:contentItem:6CMM-67Y1-JCDT-J010-00000-00&context=1519360.

31. (July 22, 2024 Monday). Concentration of particulate matter from wildfires and heat increases emergency hospitalizations. *CE Noticias Financieras English.* https://advance.lexis.com/api/document?collection=news&id=urn:contentItem:6CJB-18H1-JCG7-84WK-00000-00&context=1519360.

32. (June 27, 2024 Thursday). Panel to monitor air pollution and impacts on human health. *CE Noticias Financieras English.* https://advance.lexis.com/api/document?collection=news&id=urn:contentItem:6CC0-S2W1-DYY9-00SR-00000-00&context=1519360.

33. (June 5, 2024 Wednesday). Climate change and health: these would be the most affected populations. *CE Noticias Financieras English.* https://advance.lexis.com/api/document?collection=news&id=urn:contentItem:6C69-DCY1-JCG7-848R-00000-00&context=1519360.

34. (June 5, 2024 Wednesday). Climate change would have a greater impact on the health of pregnant women, children and the elderly.. *CE Noticias Financieras English.* https://advance.lexis.com/api/document?collection=news&id=urn:contentItem:6C69-DF01-DYY9-02PN-00000-00&context=1519360.

35. (05. Juni 2024). Soziale und ökologische Risiken für die psychische Gesundheit; forschung Es gibt zahlreiche äußere Risikofaktoren. *Nordwest-Zeitung.* https://advance.lexis.com/api/document?collection=news&id=urn:contentItem:6C62-KVJ1-F04J-30FX-00000-00&context=1519360.

36. (June 2, 2024 Sunday). Air pollution: two studies analyzed new consequences on human health. *CE Noticias Financieras English.* https://advance.lexis.com/api/document?collection=news&id=urn:contentItem:6C5N-G5C1-JCG7-800Y-00000-00&context=1519360.

37. Shivani Azad. (May 7, 2024 Tuesday). IAF choppers douse flames in Pauri. *The Times of India (TOI).* https://advance.lexis.com/api/document?collection=news&id=urn:contentItem:6BYW-P121-DXJR-H418-00000-00&context=1519360.

38. Julie Bromley. (April 10, 2024 Wednesday). Climate change adaptation is a community responsibility; If there is an unseen benefit to the global upheaval, it is watching a community rally. *The Peterborough Examiner (Ontario).* https://advance.lexis.com/api/document?collection=news&id=urn:contentItem:6BS5-MBM1-F197-502M-00000-00&context=1519360.

39. Julie Bromley. (April 10, 2024 Wednesday). GreenUP: Climate change adaptation is a community responsibility. *The Peterborough Examiner (Ontario) Online.* https://advance.lexis.com/api/document?collection=news&id=urn:contentItem:6BS5-MBM1-F197-5033-00000-00&context=1519360.

40. (April 9, 2024 Tuesday). Heatwaves and our health. *Business Mirror (Philippines).* https://advance.lexis.com/api/document?collection=news&id=urn:contentItem:6BS4-CY31-F00C-606R-00000-00&context=1519360.

41. Gabrielle Porter . (April 5, 2024 Friday). Stretching federal aid for rural health care. *The Santa Fe New Mexican.* https://advance.lexis.com/api/document?collection=news&id=urn:contentItem:6BR7-N9N1-JBSG-1005-00000-00&context=1519360.

42. (April 4, 2024 Thursday). Fires, Sleep Studies, Legislative Actions: The Week in Health. *CE Noticias Financieras English.* https://advance.lexis.com/api/document?collection=news&id=urn:contentItem:6BR3-6FD1-JCG7-839W-00000-00&context=1519360.

43. (April 4, 2024 Thursday). Valley of Mexico has only registered 8 clean days of pollutants so far in 2024. *CE Noticias Financieras English.* https://advance.lexis.com/api/document?collection=news&id=urn:contentItem:6BR3-6H21-DYY9-04S0-00000-00&context=1519360.

44. (March 29, 2024 Friday). Wildfires Threaten Public Health. *CE Noticias Financieras English.* https://advance.lexis.com/api/document?collection=news&id=urn:contentItem:6BNT-C011-DYY9-04JK-00000-00&context=1519360.

45. Bob Weber The Canadian Press. (March 25, 2024 Monday). Alberta scientists band together to shift climate change focus to health impacts. *The Hamilton Spectator (Ontario).* https://advance.lexis.com/api/document?collection=news&id=urn:contentItem:6BMS-3BC1-F197-5090-00000-00&context=1519360.

46. (March 24, 2024). Alberta scientists working to shift climate change focus to health impacts. *Abbotsford News.* https://advance.lexis.com/api/document?collection=news&id=urn:contentItem:6BMK-X791-JD2C-J00K-00000-00&context=1519360.

47. Ava Sasani. (February 26, 2024 Monday). Vast swaths of US will be exposed to polluted air by 2054, says report. *The Guardian (London).* https://advance.lexis.com/api/document?collection=news&id=urn:contentItem:6BDT-9JF1-DY4H-K35R-00000-00&context=1519360.

48. (February 21, 2024 Wednesday). Challenges ahead. *CE Noticias Financieras English.* https://advance.lexis.com/api/document?collection=news&id=urn:contentItem:6BCX-MWN1-DYY9-03YR-00000-00&context=1519360.

49. (February 9, 2024 Friday). EPA's new air pollution rules: U-M expert provides insight. *Michigan Independent: University of Michigan - Ann Arbor.* https://advance.lexis.com/api/document?collection=news&id=urn:contentItem:6B96-NN51-JBSN-303W-00000-00&context=1519360.

50. (February 9, 2024 Friday). Remaining challenges. *CE Noticias Financieras English.* https://advance.lexis.com/api/document?collection=news&id=urn:contentItem:6B9K-F191-JCG7-8148-00000-00&context=1519360.

51. Wendy Johnson. (February 8, 2024 Thursday). New Mexico must prepare for next climate emergency. *The Santa Fe New Mexican.* https://advance.lexis.com/api/document?collection=news&id=urn:contentItem:6B93-GCP1-DY7H-R00W-00000-00&context=1519360.

52. (January 27, 2024 Saturday). How pollution affects your health. *CE Noticias Financieras English.* https://advance.lexis.com/api/document?collection=news&id=urn:contentItem:6B6K-VMC1-DYY9-0512-00000-00&context=1519360.

53. (January 26, 2024 Friday). Ecoanxiety: how does the climate crisis affect mental health?. *CE Noticias Financieras English.* https://advance.lexis.com/api/document?collection=news&id=urn:contentItem:6B6C-CVT1-JCG7-81V0-00000-00&context=1519360.

54. (January 24, 2024 Wednesday). Poor air quality can cause or aggravate respiratory and even mental health illnesses. It is important to protect yourself from smoke during this season when forest fires have increased in Colombia. We tell you what you can do.. *CE Noticias Financieras English.* https://advance.lexis.com/api/document?collection=news&id=urn:contentItem:6B5Y-FS91-DYY9-029C-00000-00&context=1519360.

55. (Montag 22. Januar 2024). Der Klimawandel macht uns krank. *Kölner Express.* https://advance.lexis.com/api/document?collection=news&id=urn:contentItem:6B59-59V1-JBR8-401B-00000-00&context=1519360.

56. Michael Wood. (January 14, 2024 Sunday). The deadline to repay small-business loans must be extended; A reprieve makes more sense cost-wise than forcing many to close up. *The Vancouver Province (British Columbia).* https://advance.lexis.com/api/document?collection=news&id=urn:contentItem:6B3K-2KB1-JCDT-J1H9-00000-00&context=1519360.

57. (January 4, 2024 Thursday). June. *Daily Miner & News (Kenora).* https://advance.lexis.com/api/document?collection=news&id=urn:contentItem:6B1F-C0P1-DYWW-84Y9-00000-00&context=1519360.

58. Ross Hunter. (January 3, 2024 Wednesday). Climate change health impacts must be central to NHS future, say Scottish Greens. *The National (Scotland).* https://advance.lexis.com/api/document?collection=news&id=urn:contentItem:6B17-8F81-DY7X-T54W-00000-00&context=1519360.

59. Ben Spencer. (December 3, 2023 Sunday). Forest fires, AI and sex in space; The most exciting science books imagined the future of humanity and made us rethink our processed diets. *The Sunday Times (London).* https://advance.lexis.com/api/document?collection=news&id=urn:contentItem:69SM-9RS1-DYTY-C0CN-00000-00&context=1519360.

60. Bernie Sanders. (November 30, 2023 Thursday). Change is coming. The question is: what kind of change will it be?. *The Guardian (London).* https://advance.lexis.com/api/document?collection=news&id=urn:contentItem:69S1-CN31-JBNF-W492-00000-00&context=1519360.

61. (Montag 20. November 2023). ,,Ich habe den Eindruck, dass die Eltern uns immer auch das Sterben beibringen". *Der Tagesspiegel.* https://advance.lexis.com/api/document?collection=news&id=urn:contentItem:69NT-PP61-JCR4-R028-00000-00&context=1519360.

62. Sally Foy. (October 12, 2023 Thursday). Fearful memories fly like embers in bushfire wind. *Goulburn Post.* https://advance.lexis.com/api/document?collection=news&id=urn:contentItem:69CC-XB41-F0J6-J0MW-00000-00&context=1519360.

63. Marion Williams. (October 6, 2023 Friday). Dr Holland praises coordinated, efficient emergency response to Bermagui bushfires. *Bega District News.* https://advance.lexis.com/api/document?collection=news&id=urn:contentItem:69B4-2WW1-JD34-V0WD-00000-00&context=1519360.

64. (September 22, 2023 Friday). Pollution from forest fires is a growing global threat. *CE Noticias Financieras English.* https://advance.lexis.com/api/document?collection=news&id=urn:contentItem:697G-WR41-DYY9-0500-00000-00&context=1519360.

65. Cherki, Marc. (Jeudi 21 Septembre 2023). Les feux de forêt aggravent la pollution globale de l'air. *Le Figaro Économie.* https://advance.lexis.com/api/document?collection=news&id=urn:contentItem:6971-YW31-JBYG-200Y-00000-00&context=1519360.

66. Cherki, Marc. (Jeudi 21 Septembre 2023). Les feux de forêt aggravent la pollution globale de l'air; À cause des incendies, 2,2 milliards de personnes dans le monde respirent un air trop pollué aux particules fines au moins un jour par an.. *Le Figaro.* https://advance.lexis.com/api/document?collection=news&id=urn:contentItem:6971-YX21-DXP4-D00Y-00000-00&context=1519360.

67. (September 20, 2023 Wednesday). Learn to live with wildfire smoke, health minister tells British Columbians. *Times Colonist (Victoria, British Columbia).* https://advance.lexis.com/api/document?collection=news&id=urn:contentItem:696V-KYC1-DYWW-80RH-00000-00&context=1519360.

68. (Dienstag 12. September 2023). Klima und Gesundheit im Einklang?; Schon wieder geht es ums Klima – und das muss sein. Denn klimatische Veränderungen haben einen immensen Einfluss darauf, wie es in Zukunft um unsere Gesundheit und medizinische Versorgung bestellt ist. Von Anne Schur. *Südwest Presse.* https://advance.lexis.com/api/document?collection=news&id=urn:contentItem:6954-0CF1-JBN8-P2GF-00000-00&context=1519360.

69. Luis Fernando Espin. (September 7, 2023 Thursday). Respiratory, eye and psychological problems are treated in areas affected by fires in Quito. *CE Latin America Migration English.* https://advance.lexis.com/api/document?collection=news&id=urn:contentItem:6949-BDG1-JCG7-80TR-00000-00&context=1519360.

70. (September 6, 2023 Wednesday). Green light for the Extremadura Responde program, a psychological care service for the prevention of suicide.. *CE Noticias Financieras English.* https://advance.lexis.com/api/document?collection=news&id=urn:contentItem:6943-C7M1-DYY9-014G-00000-00&context=1519360.

71. (August 30, 2023). AHS assisting NWT evacuee patients. *Bashaw Star.* https://advance.lexis.com/api/document?collection=news&id=urn:contentItem:692F-YM81-JD2C-J0TH-00000-00&context=1519360.

72. Lauren Collins. (August 25, 2023). Doctors, nurses want B.C. to limit wildfire, climate impacts of LNG industry. *Abbotsford News.* https://advance.lexis.com/api/document?collection=news&id=urn:contentItem:691G-SS21-F0HF-8516-00000-00&context=1519360.

73. The Associated Press. (August 23, 2023 Wednesday). From Europe to Canada to Hawaii, photos capture destructive power of wildfires. *The Independent (United Kingdom).* https://advance.lexis.com/api/document?collection=news&id=urn:contentItem:6910-2XF1-DY4H-K4DR-00000-00&context=1519360.

74. Florian Gann. (Montag 21. August 2023). Wie das Klima die Psyche beeinflusst. *Schwarzwälder Bote.* https://advance.lexis.com/api/document?collection=news&id=urn:contentItem:690F-6MY1-JBF1-03BG-00000-00&context=1519360.

75. Florian Gann. (Montag 21. August 2023). Wie sich das Klima auf die Psyche auswirkt. *Schwarzwälder Bote.* https://advance.lexis.com/api/document?collection=news&id=urn:contentItem:690F-6MY1-JBF1-03BH-00000-00&context=1519360.

76. (August 19, 2023 Saturday). GOVERNOR KOTEK VISITS JOSEPHINE, CURRY, COOS COUNTIES ON ONE OREGON TOUR. *Indian eGov Newswire.* https://advance.lexis.com/api/document?collection=news&id=urn:contentItem:690N-SXV1-F11P-X178-00000-00&context=1519360.

77. David Shearman. (August 17, 2023 Thursday). We all need to watch out for each other as the world heats up. *Grey-Bruce This Week.* https://advance.lexis.com/api/document?collection=news&id=urn:contentItem:68YK-8JF1-JCDT-J1K0-00000-00&context=1519360.

78. (Dienstag 15. August 2023). 79 Prozent rauf!; Neuer Krankenstand-Rekord in Berlin; Rekord an Krankschreibungen!; Jeder Zweite meldete sich in diesem Jahr schon ab. "Bei vielen Deutschen ist der Akku leer", sagen Experten. *B.Z..* https://advance.lexis.com/api/document?collection=news&id=urn:contentItem:68Y5-J1W1-DY2B-S297-00000-00&context=1519360.

79. Ralf Klostermann, Jenna Müller. (Dienstag 15. August 2023). Rekord: Jeder 2 . meldete sichab!; Deutschland schreibt sich KRANK; Rekord an Krankschreibungen!; Jeder Zweite meldete sich in diesem Jahr schon ab. "Bei vielen Deutschen ist der Akku leer", sagen Experten. *BILD Bund.* https://advance.lexis.com/api/document?collection=news&id=urn:contentItem:68Y5-J1W1-DY2B-S1WY-00000-00&context=1519360.

80. (August 14, 2023 Monday). [World leaders have to pull us out of 'the mad ages']. *The Chronicle Journal.* https://advance.lexis.com/api/document?collection=news&id=urn:contentItem:6901-W6S1-DYWW-84BM-00000-00&context=1519360.

81. (Samstag 5. August 2023). Seit Corona leben die Menschen in der Dauer-Krise. *Frankenpost.* https://advance.lexis.com/api/document?collection=news&id=urn:contentItem:68W1-NRD1-DXX2-P256-00000-00&context=1519360.

82. dpa Von Christoph Driessen. (Montag 31. Juli 2023). Gesellschaft; Gesellschaft Krisen und kein Ende - Die erschöpfte Republik. *Weiler Zeitung.* https://advance.lexis.com/api/document?collection=news&id=urn:contentItem:6925-01M1-F134-01VT-

00000-00&context=1519360.

83. (July 28, 2023 Friday). More to mental health around bushfires. *Canberra City News.* https://advance.lexis.com/api/document?collection=news&id=urn:contentItem:68T9-23N1-JBJ5-X18F-00000-00&context=1519360.

84. (July 27, 2023 Thursday). Faced with heat waves in Europe, WHO recommends using common sense. *CE Noticias Financieras English.* https://advance.lexis.com/api/document?collection=news&id=urn:contentItem:68TB-JF71-JCG7-81G7-00000-00&context=1519360.

85. (Jeudi 27 Juillet 2023). FEUX DE FORÊT : La situation est sous contrôle. *Le Courrier d'Algerie.* https://advance.lexis.com/api/document?collection=news&id=urn:contentItem:68T5-BFH1-JDJN-6374-00000-00&context=1519360.

86. (July 26, 2023 Wednesday). Extreme heat and forest fires can trigger all kinds of diseases: WHO. *CE Noticias Financieras English.* https://advance.lexis.com/api/document?collection=news&id=urn:contentItem:68T4-R9N1-DYY9-00DJ-00000-00&context=1519360.

87. Dr. Peter Nieman. (July 22, 2023 Saturday). Deep climate change divisions can lead to losing sight of its effects on children; Important to educate kids using the facts from reliable, credible scientific resources. *The Calgary Herald (Alberta).* https://advance.lexis.com/api/document?collection=news&id=urn:contentItem:68S2-HJW1-JBKR-C2RG-00000-00&context=1519360.

88. Michele Jarvie. (July 22, 2023 Saturday). Nieman: Deep divisions over climate change leads to losing sight of impact on children. *Postmedia Breaking News.* https://advance.lexis.com/api/document?collection=news&id=urn:contentItem:68S3-H771-F125-108W-00000-00&context=1519360.

89. Manuela Vega. (June 28, 2023 Wednesday). Toronto's air quality is among the worst in the world - again - due to wildfire smoke. *The Star (Toronto, Ontario) Online.* https://advance.lexis.com/api/document?collection=news&id=urn:contentItem:68K1-SG01-F197-526S-00000-00&context=1519360.

90. Manuela Vega. (June 28, 2023 Wednesday). Toronto's air quality is officially the worst in the world due to wildfire smoke. *thestar.com.* https://advance.lexis.com/api/document?collection=news&id=urn:contentItem:68K1-T3F1-JDV5-F483-00000-00&context=1519360.

91. (June 22, 2023 Thursday). How prolonged exposure to wildfire smoke could affect your health. *CE Noticias Financieras English.* https://advance.lexis.com/api/document?collection=news&id=urn:contentItem:68HW-K191-DYY9-02F1-00000-00&context=1519360.

92. (June 18, 2023 Sunday). 'Harmful': Environment Canada puts Mississauga and Brampton under Special Air Quality Statement warning of high levels of wildfire smoke pollution. *Mississauga News.* https://advance.lexis.com/api/document?collection=news&id=urn:contentItem:68H2-7TT1-JDV5-F113-00000-00&context=1519360.

93. Jacey Fortin. (June 9, 2023 Friday). With pandemic fears fresh, airborne anxieties are nothing new.. *The New York Times* . https://advance.lexis.com/api/document?collection=news&id=urn:contentItem:68DY-0591-DXY4-X2X7-00000-00&context=1519360.

94. Kevin Jiang. (May 16, 2023 Tuesday). Wildfires made Calgary the most polluted city on Earth. Here's how air pollution harms our health. *thestar.com.*

https://advance.lexis.com/api/document?collection=news&id=urn:contentItem:6881-6T31-JDV5-F062-00000-00&context=1519360.

95. (May 2, 2023 Tuesday). UFRJ will gather researchers to discuss inequalities. *CE Noticias Financieras English.* https://advance.lexis.com/api/document?collection=news&id=urn:contentItem:6851-2TV1-DYY9-03DV-00000-00&context=1519360.

96. (March 26, 2023 Sunday). What is eco-anxiety: learn more about the fear of the consequences of climate change?. *CE Noticias Financieras English.* https://advance.lexis.com/api/document?collection=news&id=urn:contentItem:67W4-55Y1-JCG7-81CV-00000-00&context=1519360.

97. (March 24, 2023 Friday). Health recommendations guide for the Alto Mijares Fire. *CE Noticias Financieras English.* https://advance.lexis.com/api/document?collection=news&id=urn:contentItem:67VP-7NK1-JCG7-80W3-00000-00&context=1519360.

98. (March 21, 2023 Tuesday). Lush forests mean healthy people. *The Bangkok Post (Thailand).* https://advance.lexis.com/api/document?collection=news&id=urn:contentItem:67V1-T2K1-F00C-645M-00000-00&context=1519360.

99. (March 21, 2023 Tuesday). Why we need healthy forests for healthy people. *CE Noticias Financieras English.* https://advance.lexis.com/api/document?collection=news&id=urn:contentItem:67V2-BB31-JCG7-81K8-00000-00&context=1519360.

100. (February 9, 2023 Thursday). Online psychology startup offers free care to fire victims. *CE Noticias Financieras English.* https://advance.lexis.com/api/document?collection=news&id=urn:contentItem:67HH-NBR1-DYY9-0082-00000-00&context=1519360.

101. Nick Knight. (February 7, 2023). Firefighters battle wildfire in Dartmoor Forest. *Okehampton Times.* https://advance.lexis.com/api/document?collection=news&id=urn:contentItem:683R-MFB1-JCMC-W29X-00000-00&context=1519360.

102. (February 2, 2023). UVic study finds traffic pollution can impair brain function. *Peninsula News Review.* https://advance.lexis.com/api/document?collection=news&id=urn:contentItem:67G0-8N31-F0HF-847Y-00000-00&context=1519360.

103. (January 25, 2023). Traffic pollution can impair brain function, say B.C. researchers. *Abbotsford News.* https://advance.lexis.com/api/document?collection=news&id=urn:contentItem:67DG-NF71-JD2C-J3XS-00000-00&context=1519360.

104. (November 15, 2022 Tuesday). What are the origins of the term 'climate anxiety'?. *Malay Mail.* https://advance.lexis.com/api/document?collection=news&id=urn:contentItem:66W1-F421-F12F-F092-00000-00&context=1519360.

105. Susanne Schwarz. (Mittwoch 26. Oktober 2022). Klimakrise bedroht globale Gesundheit; Wissenschaftler:innen warnen vor weltweit mehr Kranken, Hitzetoten, Hunger und Mangelernährung. *taz, die tageszeitung.* https://advance.lexis.com/api/document?collection=news&id=urn:contentItem:66PH-T211-F117-G1XM-00000-00&context=1519360.

106. (October 25, 2022 Tuesday). Governments and companies continue to pursue policies that threaten health, says Lancet. *CE Noticias Financieras English.* https://advance.lexis.com/api/document?collection=news&id=urn:contentItem:66PP-K701-JCG7-838F-00000-00&context=1519360.

107. (October 13, 2022 Thursday). Disease, pollution and fires: how climate change is robbing us of life. *CE Noticias Financieras English.*

https://advance.lexis.com/api/document?collection=news&id=urn:contentItem:66M4-XY01-DYY9-02BT-00000-00&context=1519360.

108. (September 21, 2022 Wednesday). Santa Cruz defying its own future. *CE Noticias Financieras English.* https://advance.lexis.com/api/document?collection=news&id=urn:contentItem:66FF-JYX1-DYY9-01FV-00000-00&context=1519360.

109. (September 18, 2022 Sunday). Climate emergency means public health crisis. *CE Noticias Financieras English.* https://advance.lexis.com/api/document?collection=news&id=urn:contentItem:66DT-MW41-DYY9-043D-00000-00&context=1519360.

110. (September 1, 2022). Climate activism is good for your health. *Sooke News Mirror.* https://advance.lexis.com/api/document?collection=news&id=urn:contentItem:6692-K6D1-F0HF-83KN-00000-00&context=1519360.

111. David Suzuki. (August 30, 2022 Tuesday). Climate activism is good for health; Science Matters. *Sherbrooke Record (Quebec).* https://advance.lexis.com/api/document?collection=news&id=urn:contentItem:66F6-X3V1-DYWX-Y40W-00000-00&context=1519360.

112. (Dienstag 30. August 2022). »Man scrollt und scrollt«; Für viele gehören Nachrichten zum Alltag. Manche Menschen leiden jedoch unter ihrem exzessiven News-Konsum.. *Aachener Zeitung.* https://advance.lexis.com/api/document?collection=news&id=urn:contentItem:668J-38W1-F154-40F3-00000-00&context=1519360.

113. (Montag 29. August 2022). «Man scrollt und scrollt» - Was macht exzessiver News-Konsum mit uns?; Medienkompetenz Ein nicht unbeachtlicher Teil der Menschen weist laut einer US-Studie einen problematischen Nachrichtenkonsum auf. Dies könnte sich möglicherweise negativ auf deren Gesundheit auswirken.. *Liechtensteiner Volksblatt.* https://advance.lexis.com/api/document?collection=news&id=urn:contentItem:668B-0BV1-DYY8-D16P-00000-00&context=1519360.

114. (August 26, 2022). Climate activism is good for health. *Yukon News.* https://advance.lexis.com/api/document?collection=news&id=urn:contentItem:6680-NNH1-F0HF-83VS-00000-00&context=1519360.

115. (August 17, 2022 Wednesday). Air. *CE Noticias Financieras English.* https://advance.lexis.com/api/document?collection=news&id=urn:contentItem:6660-K871-JCG7-84C4-00000-00&context=1519360.

116. Alex Brown. (August 12, 2022 Friday). Public Health Agencies Lack Money to Combat Climate Threats. *Stateline.org.* https://advance.lexis.com/api/document?collection=news&id=urn:contentItem:665M-7HR1-JBTP-M10F-00000-00&context=1519360.

117. (July 23, 2022 Saturday). Heat waves to continue until 2060 due to climate change. *CE Noticias Financieras English.* https://advance.lexis.com/api/document?collection=news&id=urn:contentItem:660N-T351-DYY9-0298-00000-00&context=1519360.

118. Deborah Sullivan Brennan. (June 29, 2022 Wednesday). County supes approve $7.36B budget; Behavioral health needs, homeless, environmental issues get funding boost. *The San Diego Union-Tribune.* https://advance.lexis.com/api/document?collection=news&id=urn:contentItem:65TK-8WD1-DXXV-300R-00000-00&context=1519360.

119. CLAIRE RUSH Associated Press/ Report for America. (June 18, 2022 Saturday). Report: Fires, heat waves spur 'climate anxiety' in youth. *The Columbian (Vancouver, Washington).* https://advance.lexis.com/api/document?collection=news&id=urn:contentItem:65R1-C2H1-JBSN-04FH-

00000-00&context=1519360.

120. (June 8, 2022). Air pollution is detrimental to everyone. *Comox Valley Record.* https://advance.lexis.com/api/document?collection=news&id=urn:contentItem:65MY-20V1-JD2C-J1KT-00000-00&context=1519360.

121. (June 7, 2022). OUR PLANETARY HEALTH: Air pollution is detrimental to everyone. *Comox Valley Record.* https://advance.lexis.com/api/document?collection=news&id=urn:contentItem:65MR-23F1-F0HF-803R-00000-00&context=1519360.

122. (May 22, 2022 Sunday). CHANGING MENTAL climate. *The Pioneer (India).* https://advance.lexis.com/api/document?collection=news&id=urn:contentItem:65H5-N2C1-JDKC-R097-00000-00&context=1519360.

123. (May 11, 2022 Wednesday). Pollution in CDMX takes its toll on young people; damage already evident. *CE Noticias Financieras English.* https://advance.lexis.com/api/document?collection=news&id=urn:contentItem:65F3-F571-DYY9-02HY-00000-00&context=1519360.

124. Scott Wyland . (May 3, 2022 Tuesday). Changing breezes advance fire closer, stymie crews. *The Santa Fe New Mexican.* https://advance.lexis.com/api/document?collection=news&id=urn:contentItem:65CB-55B1-DY7H-R0KW-00000-00&context=1519360.

125. Toyin Adebayo. (April 7, 2022 Thursday). Environmental Crisis Worsening Non-communicable Diseases, Others – FG. *Daily Independent (Nigeria).* https://advance.lexis.com/api/document?collection=news&id=urn:contentItem:655P-CNW1-F11P-X146-00000-00&context=1519360.

126. (April 7, 2022 Thursday). To develop equitably, address five priorities. *Hindustan Times Lucknow.* https://advance.lexis.com/api/document?collection=news&id=urn:contentItem:655G-S1M1-JDKC-R1RW-00000-00&context=1519360.

127. Express News Service. (April 6, 2022 Wednesday). Prioritise equitable health to build well-being societies for a healthier, fairer, greener world: WHO. *Indian Express.* https://advance.lexis.com/api/document?collection=news&id=urn:contentItem:655D-SYY1-DXMP-K233-00000-00&context=1519360.

128. Himalayan News Service. (April 6, 2022 Wednesday). WHO calls for prioritising equitable health for healthier, fairer, greener world. *Himalayan Times.* https://advance.lexis.com/api/document?collection=news&id=urn:contentItem:655N-DBR1-DXMP-K181-00000-00&context=1519360.

129. (Samstag 2. April 2022). Kranke Erde - kranker Körper. *Kronen Zeitung.* https://advance.lexis.com/api/document?collection=news&id=urn:contentItem:654T-2XX1-DYY8-D0KN-00000-00&context=1519360.

130. (March 14, 2022 Monday). Jorge Manjarrez: Solastalgia. *CE Noticias Financieras English.* https://advance.lexis.com/api/document?collection=news&id=urn:contentItem:650R-4D01-DYY9-02VW-00000-00&context=1519360.

131. (March 6, 2022 Sunday). More diseases and deaths due to climate change. *CE Noticias Financieras English.* https://advance.lexis.com/api/document?collection=news&id=urn:contentItem:64Y1-H2T1-DYY9-00JC-00000-00&context=1519360.

132. Excélsior. (March 6, 2022 Sunday). More diseases and deaths from climate change. *CE Latin America Migration English.* https://advance.lexis.com/api/document?collection=news&id=urn:contentItem:64Y1-

H2S1-DYY9-045J-00000-00&context=1519360.

133. (February 22, 2022 Tuesday). Noise, the pollution that (literally) keeps you awake at night. *CE Noticias Financieras English.* https://advance.lexis.com/api/document?collection=news&id=urn:contentItem:64VF-WTV1-JCG7-84K7-00000-00&context=1519360.

134. (February 18, 2022 Friday). Hearing pollution threatens public health: UN. *CE Noticias Financieras English.* https://advance.lexis.com/api/document?collection=news&id=urn:contentItem:64TM-0T91-DYY9-02Y9-00000-00&context=1519360.

135. Samuel Webb. (February 17, 2022 Thursday). Deadly wildfires and noise pollution among critical environmental threats, UN warns. *The Independent (United Kingdom).* https://advance.lexis.com/api/document?collection=news&id=urn:contentItem:64T7-CDH1-JBNF-W44B-00000-00&context=1519360.

136. (February 17, 2022 Thursday). UN report highlights threats to the environment. *CE Noticias Financieras English.* https://advance.lexis.com/api/document?collection=news&id=urn:contentItem:64TD-28Y1-DYY9-02TT-00000-00&context=1519360.

137. (February 17, 2022 Thursday). UN warns of threat from noise pollution and wildfires. *CE Noticias Financieras English.* https://advance.lexis.com/api/document?collection=news&id=urn:contentItem:64TD-2931-JCG7-808J-00000-00&context=1519360.

138. MYK. (February 17, 2022). PNUE - La pollution sonore dans les villes un danger croissant pour la santé publique et pour les animaux (Rapport). *Tunis Afrique Presse.* https://advance.lexis.com/api/document?collection=news&id=urn:contentItem:64TD-H8Y1-JBJ4-24J8-00000-00&context=1519360.

139. (February 9, 2022 Wednesday). Preview of "El tercer inconsciente", by Franco "Bifo" Berardi. *CE Noticias Financieras English.* https://advance.lexis.com/api/document?collection=news&id=urn:contentItem:64RP-8501-DYY9-04BB-00000-00&context=1519360.

140. BILL POEHLER The Salem Statesman Journal. (February 7, 2022 Monday). Fire survivors report health issues. *The Columbian (Vancouver, Washington).* https://advance.lexis.com/api/document?collection=news&id=urn:contentItem:64R3-BJD1-JBSN-0080-00000-00&context=1519360.

141. Tiffany Crawford. (January 2, 2022 Sunday). The burden of eco-anxiety; Climate-related disasters are causing surge in mental-health problems in B.C., physician says. *The Vancouver Province (British Columbia).* https://advance.lexis.com/api/document?collection=news&id=urn:contentItem:64FC-WJV1-DY2S-T51X-00000-00&context=1519360.

142. Tiffany Crawford. (January 1, 2022 Saturday). Heat dome, wildfires, floods: Climate-related disasters causing surge in mental health problems in B.C., physician. *Postmedia Breaking News.* https://advance.lexis.com/api/document?collection=news&id=urn:contentItem:64F6-20J1-JDK3-929H-00000-00&context=1519360.

143. Julia Giertz, Dpa. (December 13, 2021 Monday). The inflation of the term "apocalypse; Psychologists deal with the fear of the future. *Die Welt (English).* https://advance.lexis.com/api/document?collection=news&id=urn:contentItem:6493-8VK1-DY2B-S0GT-00000-00&context=1519360.

144. Julia Giertz, Dpa. (Montag 13. Dezember 2021). Die Inflation des Begriffs "Apokalypse"; Psychologen beschäftigen sich mit der Zukunfts-Angst. *Die Welt.* https://advance.lexis.com/api/document?collection=news&id=urn:contentItem:6493-8VM1-DY2B-S11P-00000-00&context=1519360.

145. (Montag 13. Dezember 2021). Die ständige Angst vor der Apokalypse. *Frankenpost.* https://advance.lexis.com/api/document?collection=news&id=urn:contentItem:6493-B221-DXX2-P247-00000-00&context=1519360.

146. (Montag 13. Dezember 2021). Kommt die Apokalypse?; Vom Weltuntergang wurde schon immer geredet. Zuversicht trotz Pandemie und Klimawandel. *General-Anzeiger (Bonn).* https://advance.lexis.com/api/document?collection=news&id=urn:contentItem:6493-48P1-JC45-703C-00000-00&context=1519360.

147. (December 3, 2021). B.C. adds paramedics, dispatchers, increasing treatment options. *Abbotsford News.* https://advance.lexis.com/api/document?collection=news&id=urn:contentItem:6474-NWP1-F0HF-8139-00000-00&context=1519360.

148. Dr. Danielle Martin And Dr. Samantha Green. (November 18, 2021 Thursday). The intersection of health and climate. *Waterloo Region Record (Ontario).* https://advance.lexis.com/api/document?collection=news&id=urn:contentItem:643S-SY41-JDV5-F2FS-00000-00&context=1519360.

149. scott.huish. (November 15, 2021 Monday). 2nd Place Affordable Housing - Red Rock Creek Commons. *Daily Journal of Commerce.* https://advance.lexis.com/api/document?collection=news&id=urn:contentItem:6440-4831-F053-W1SC-00000-00&context=1519360.

150. Danielle Martin And Samantha Green Contributors Dr. Danielle Martin Is A Family Physician And Chair Of The Department Of Family And Community Medicine At U Of T.dr. Samantha Green Is A Family Physician And Faculty Lead In Climate Change And Heal. (November 15, 2021 Monday). Health sector must help fight. *The Toronto Star.* https://advance.lexis.com/api/document?collection=news&id=urn:contentItem:6434-VC21-JDV5-F0NJ-00000-00&context=1519360.

151. Steve Dow. (November 11, 2021 Thursday). The Last Woman in the World by Inga Simpson review - apocalyptic thriller preys on Australians' worst nightmares. *The Guardian (London).* https://advance.lexis.com/api/document?collection=news&id=urn:contentItem:642B-7331-DY4H-K02D-00000-00&context=1519360.

152. Ching Ann Hui and Woo Qiyun For The Straits Times. (November 8, 2021 Monday). When climate change affects human health; Zoonotic diseases, heatwaves and wildfires already posing health risks. *The Straits Times (Singapore).* https://advance.lexis.com/api/document?collection=news&id=urn:contentItem:641K-FXT1-DYX4-01VJ-00000-00&context=1519360.

153. (October 27, 2021 Wednesday). 5 Cabinet takeaways. *National Post (f/k/a The Financial Post) (Canada).* https://advance.lexis.com/api/document?collection=news&id=urn:contentItem:63Y2-GPB1-JBKR-G28M-00000-00&context=1519360.

154. (October 26, 2021 Tuesday). Five things to know about Prime Minister Justin Trudeau's new cabinet. *National Post (f/k/a The Financial Post) (Canada).* https://advance.lexis.com/api/document?collection=news&id=urn:contentItem:63XY-G001-DY2T-615T-00000-00&context=1519360.

155. Audrey Tan. (October 13, 2021 Wednesday). Cut emissions faster, health sector urges. *The Straits Times (Singapore).* https://advance.lexis.com/api/document?collection=news&id=urn:contentItem:63V0-1MD1-JCF2-819C-00000-00&context=1519360.

156. (October 12, 2021 Tuesday). Disasters increase fivefold in 50 years around the world. *CE Noticias Financieras English.* https://advance.lexis.com/api/document?collection=news&id=urn:contentItem:63V3-K7V1-JBJN-M3M8-00000-00&context=1519360.

157. (October 12, 2021 Tuesday). Reduce emissions faster to safeguard human health, healthcare professionals urge in open letter. *The Daily Star, Dhaka, Bangladesh / Asia News Network.* https://advance.lexis.com/api/document?collection=news&id=urn:contentItem:63V3-J771-JBTP-M1BC-00000-00&context=1519360.

158. Leah Gerber Waterloo Region Record Leah Gerber Is A Waterloo Region-based General Assignment Reporter For The Record. Reach Her . (September 28, 2021 Tuesday). Province to aid mental health program in farming; Minister announces Ontario is providing $380,000 to expand a resource tailored to agricultural community, veterinarians, feed sellers and more. *Waterloo Region Record (Ontario).* https://advance.lexis.com/api/document?collection=news&id=urn:contentItem:63PX-30Y1-F197-53H9-00000-00&context=1519360.

159. Leah Gerber. (September 27, 2021 Monday). Mental health in farming communities to receive more provincial support. *The Record (Waterloo Region, Ontario) Online.* https://advance.lexis.com/api/document?collection=news&id=urn:contentItem:63PS-D061-F197-5267-00000-00&context=1519360.

160. Anthony Bunn. (September 27, 2021 Monday). Senators to hear of bushfire fallout. *The Border Mail.* https://advance.lexis.com/api/document?collection=news&id=urn:contentItem:63PH-T4N1-F0J6-J1NS-00000-00&context=1519360.

161. Allison Pearson. (September 22, 2021 Wednesday). The cancer backlog is a scandal - and it's even worse than you think. *The Daily Telegraph (London).* https://advance.lexis.com/api/document?collection=news&id=urn:contentItem:63NK-HG51-DYTY-C1S5-00000-00&context=1519360.

162. (September 17, 2021 Friday). How mental health is fast becoming a casualty of climate change. *Naija 247 News.* https://advance.lexis.com/api/document?collection=news&id=urn:contentItem:63MT-JBK1-JCH9-G4CB-00000-00&context=1519360.

163. (September 16, 2021 Thursday). Mental Health Could Be the Next Casualty of Global Warming. *National Post (f/k/a The Financial Post) (Canada).* https://advance.lexis.com/api/document?collection=news&id=urn:contentItem:63MC-58K1-JBKR-G2V9-00000-00&context=1519360.

164. (September 12, 2021 Sunday). Climate change has a worrying impact on health. *CE Noticias Financieras English.* https://advance.lexis.com/api/document?collection=news&id=urn:contentItem:63KP-FGH1-JBJN-M358-00000-00&context=1519360.

165. (September 8, 2021 Wednesday). Make bushfire submission. *Gloucester Advocate.* https://advance.lexis.com/api/document?collection=news&id=urn:contentItem:63JG-B661-F0J6-J097-00000-00&context=1519360.

166. (September 7, 2021 Tuesday). This is how global warming will cause deaths beyond hurricanes and wildfires. *CE Noticias Financieras English.* https://advance.lexis.com/api/document?collection=news&id=urn:contentItem:63JM-M0G1-JBJN-M0NB-00000-00&context=1519360.

167. (September 4, 2021 Saturday). Readers write letters to the editor. *The Peterborough Examiner (Ontario).* https://advance.lexis.com/api/document?collection=news&id=urn:contentItem:63HS-SN11-F197-53NY-00000-00&context=1519360.

168. (Donnerstag 2. September 2021). Positiv bleiben. *Frankenpost.* https://advance.lexis.com/api/document?collection=news&id=urn:contentItem:63HB-55H1-JBF1-03JX-00000-00&context=1519360.

Bibliography

169. (August 31, 2021 Tuesday). Coronial inquiry into Black Summer bushfires begins. *Great Lakes Advocate.* https://advance.lexis.com/api/document?collection=news&id=urn:contentItem:63GS-GDT1-F0J6-J23J-00000-00&context=1519360.

170. Regine Warth. (Dienstag 31. August 2021). ,,Schlechte Nachrichten bereiten uns Stress". *Stuttgarter Zeitung.* https://advance.lexis.com/api/document?collection=news&id=urn:contentItem:63GX-9H91-JBYJ-90NX-00000-00&context=1519360.

171. Paul Devine Bottone. (August 30, 2021 Monday). Let's speak for the trees; Planting them can help Philadelphia and our nation address climate change and the threat it imposes to public health.. *The Philadelphia Inquirer.* https://advance.lexis.com/api/document?collection=news&id=urn:contentItem:63GR-5CY1-DYJT-20X8-00000-00&context=1519360.

172. (August 27, 2021). Wildfires take toll across region. *100 Mile House Free Press.* https://advance.lexis.com/api/document?collection=news&id=urn:contentItem:63G5-9WJ1-JD2C-J4T5-00000-00&context=1519360.

173. 100 Mile Free Press. (August 25, 2021). CRD EOC under stress due to natural disasters. *Quesnel Cariboo Observer.* https://advance.lexis.com/api/document?collection=news&id=urn:contentItem:63FR-D231-F0HF-83T2-00000-00&context=1519360.

174. (August 24, 2021 Tuesday). Latest Minnesota news, sports, business and entertainment at 3:20 p.m. CDT. *Times Colonist (Victoria, British Columbia).* https://advance.lexis.com/api/document?collection=news&id=urn:contentItem:63FM-0RN1-JBKR-9431-00000-00&context=1519360.

175. (August 16, 2021 Monday). We're all fine. *CE Noticias Financieras English.* https://advance.lexis.com/api/document?collection=news&id=urn:contentItem:63CY-7V41-JBJN-M32X-00000-00&context=1519360.

176. (August 12, 2021). Life imitates art - but the ending is not fully written. *The New Zealand Herald.* https://advance.lexis.com/api/document?collection=news&id=urn:contentItem:63BP-M2D1-JDK8-V3WW-00000-00&context=1519360.

177. Peter Moon. (August 6, 2021 Friday). Canadian rangers cope with mental stresses during COVID. *Timmins Daily Press.* https://advance.lexis.com/api/document?collection=news&id=urn:contentItem:639K-25X1-DYM8-G28Y-00000-00&context=1519360.

178. (August 1, 2021 Sunday). When climate change is also a health problem. *CE Noticias Financieras English.* https://advance.lexis.com/api/document?collection=news&id=urn:contentItem:638R-NP81-JBJN-M1GD-00000-00&context=1519360.

179. Staff. (July 16, 2021 Friday). AROUND THE WORLD IN 80 SECONDS. *The Atlanta Journal-Constitution.* https://advance.lexis.com/api/document?collection=news&id=urn:contentItem:6354-J9Y1-DYR7-J2JJ-00000-00&context=1519360.

180. The Canadian Press. (June 10, 2021). Prepare for the worst: 10 steps to get ready for wildfire smoke. *Barriere Star Journal (N. Thompson).* https://advance.lexis.com/api/document?collection=news&id=urn:contentItem:62WN-93D1-JD2C-J2BV-00000-00&context=1519360.

181. Andy Gregory. (May 4, 2021 Tuesday). Air pollution may impair memory and verbal fluency in older men, study suggests. *The Independent (United Kingdom).* https://advance.lexis.com/api/document?collection=news&id=urn:contentItem:62KH-0SM1-JBNF-W23C-00000-00&context=1519360.

182. (April 18, 2021 Sunday). DO MORE THIS EARTH DAY FOR CLIMATE. *Wisconsin State Journal (Madison, Wisconsin).* https://advance.lexis.com/api/document?collection=news&id=urn:contentItem:62G5-83G1-DY37-53S1-00000-00&context=1519360.

183. (April 7, 2021 Wednesday). Forest fires worsen air pollution, elderly finding it difficult to breathe in smoke-filled hills. *The Times of India (TOI).* https://advance.lexis.com/api/document?collection=news&id=urn:contentItem:62CN-53K1-DXJR-H532-00000-00&context=1519360.

184. Steve Dow. (March 28, 2021 Sunday). Out of the shadows. *The Sydney Morning Herald.* https://advance.lexis.com/api/document?collection=news&id=urn:contentItem:629G-4J51-F0J6-J1V8-00000-00&context=1519360.

185. (March 14, 2021 Sunday). Government to send more COVID-19 vaccines to Chubut. *CE Noticias Financieras English.* https://advance.lexis.com/api/document?collection=news&id=urn:contentItem:626W-S441-DY1R-B369-00000-00&context=1519360.

186. (March 14, 2021 Sunday). Patagonia Fires: Ministry of Health to send vaccines and make mental health team available. *CE Noticias Financieras English.* https://advance.lexis.com/api/document?collection=news&id=urn:contentItem:626W-S441-DY1R-B343-00000-00&context=1519360.

187. THARANYA ARUMUGAM. (March 8, 2021 Monday). â€˜HAZE MAY RAISE RISK OF COVID-19 COMPLICATIONSâ€™. *New Straits Times (Malaysia).* https://advance.lexis.com/api/document?collection=news&id=urn:contentItem:625B-WF81-DYR7-32VD-00000-00&context=1519360.

188. (February 23, 2021 Tuesday). One year of online containment. *CE Noticias Financieras English.* https://advance.lexis.com/api/document?collection=news&id=urn:contentItem:622V-GK41-DY1R-B450-00000-00&context=1519360.

189. Tara Deschamps. (February 20, 2021 Saturday). Ottawa urged to speed up tech bill as Facebook retaliates against Australian law. *Medicine Hat News.* https://advance.lexis.com/api/document?collection=news&id=urn:contentItem:6220-D1M1-DYKX-029W-00000-00&context=1519360.

190. Daily News Egypt. (January 26, 2021 Tuesday). Opinion| Mutating coronavirus: reaching herd immunity just got harder, but there is still hope. *Daily News Egypt.* https://advance.lexis.com/api/document?collection=news&id=urn:contentItem:61VN-YH31-JDJN-61DC-00000-00&context=1519360.

191. Tatiana Schlossberg. (January 22, 2021 Friday). Three Books Offer New Ways to Think About Environmental Disaster; The Shortlist. *The New York Times* . https://advance.lexis.com/api/document?collection=news&id=urn:contentItem:61TV-RBS1-JBG3-63JV-00000-00&context=1519360.

192. Bernie Sanders. (January 20, 2021 Wednesday). Joe Biden must put an end to business as usual. Here's where to start. *The Guardian (London).* https://advance.lexis.com/api/document?collection=news&id=urn:contentItem:61TD-59C1-JBNF-W43T-00000-00&context=1519360.

193. (mercredi 13 janvier 2021). Si l'on ne fait rien, le taux d'incidence va augmenter. *Le Courrier Picard.* https://advance.lexis.com/api/document?collection=news&id=urn:contentItem:61RW-0MV1-F035-T116-00000-00&context=1519360.

194. Elliot Williams. (January 3, 2021 Sunday). Silver linings among the dark clouds of Mogo. *Canberra Times (Australia).* https://advance.lexis.com/api/document?collection=news&id=urn:contentItem:61NK-9R61-F0J6-J3P7-00000-00&context=1519360.

195. Jackie Armstrong-Homeniuk. (December 31, 2020 Thursday). Reflecting on 2020. *Fort Saskatchewan Record.* https://advance.lexis.com/api/document?collection=news&id=urn:contentItem:61N3-R711-JC4F-W3V4-00000-00&context=1519360.

196. Ian Randall For Mailonline. (December 7, 2020 Monday). Wildfires that ravaged California in 2018 cost the US economy almost $150 BILLION in damages. *MailOnline.* https://advance.lexis.com/api/document?collection=news&id=urn:contentItem:61G1-Y171-JBNF-W489-00000-00&context=1519360.

197. (December 1, 2020 Tuesday). Health.-Forest fires, cars and power plants join the list of Alzheimer's risk factors. *CE Noticias Financieras English.* https://advance.lexis.com/api/document?collection=news&id=urn:contentItem:61DX-XT61-JBJN-M4ST-00000-00&context=1519360.

198. By Ariella Cook-Shonkoff. (November 28, 2020 Saturday). Here to Help; How Parents Can Tame-The Stress of Climate Crisis. *The New York Times.* https://advance.lexis.com/api/document?collection=news&id=urn:contentItem:61D2-P6Y1-DXY4-X3FY-00000-00&context=1519360.

199. (November 26, 2020 Thursday). NHS mental health absences rose a fifth. *Oxford Mail.* https://advance.lexis.com/api/document?collection=news&id=urn:contentItem:61CM-K4V1-F0JC-M144-00000-00&context=1519360.

200. Ariella Cook-Shonkoff. (November 17, 2020 Tuesday). How Parents Can Tame the Stress of Climate Crises. *The New York Times* . https://advance.lexis.com/api/document?collection=news&id=urn:contentItem:619R-BGB1-DXY4-X1G0-00000-00&context=1519360.

201. ROBERT OLSON. (October 11, 2020 Sunday). Sleep, faith, breathing are ways to combat hopelessness [Senior Life column]. *LNP (Lancaster, PA).* https://advance.lexis.com/api/document?collection=news&id=urn:contentItem:611W-D2T1-DYRG-S33B-00000-00&context=1519360.

202. (September 30, 2020 Wednesday). Youth, anglos, health staff hit hard by pandemic anxiety: study. *The Gazette (Montreal).* https://advance.lexis.com/api/document?collection=news&id=urn:contentItem:60YG-7N91-DY2T-C04W-00000-00&context=1519360.

203. (September 29, 2020 Tuesday). Coronavirus live updates: Youth, anglophones, health workers most affected by pandemic anxiety - study. *Postmedia Breaking News.* https://advance.lexis.com/api/document?collection=news&id=urn:contentItem:60Y9-XF11-F125-102N-00000-00&context=1519360.

204. Derek Chen. (September 29, 2020 Tuesday). Research Roundup: COVID impacts teens' mental health, CAT scans predict wildfires, news covers non-white homicide victims less. *The Stanford Daily: Stanford University.* https://advance.lexis.com/api/document?collection=news&id=urn:contentItem:60Y8-8F51-JBSN-34N1-00000-00&context=1519360.

205. (September 25, 2020 Friday). Jocelyn's Jottings: Dealing with anxious feelings a reminder to take care. *Revelstoke Review.* https://advance.lexis.com/api/document?collection=news&id=urn:contentItem:60XN-VW91-JDH1-C31B-00000-00&context=1519360.

206. (September 22, 2020 Tuesday). Mall walking excellent, safe workout for all. *The Columbian (Vancouver, Washington).* https://advance.lexis.com/api/document?collection=news&id=urn:contentItem:613T-1WD1-DY7P-P18X-00000-00&context=1519360.

207. (September 15, 2020 Tuesday). Editorial: It's okay to give yourself some grace Try these fun and funky Friday night plans Try these fun and funky Friday night. *Montana Kaimin: University of Montana.* https://advance.lexis.com/api/document?collection=news&id=urn:contentItem:60VC-9B91-DY7P-T1HW-00000-00&context=1519360.

208. Nick Pearce. (September 8, 2020 Tuesday). Slow wildfire season a relief for northern Saskatchewan. *The Star Phoenix (Saskatoon, Saskatchewan).* https://advance.lexis.com/api/document?collection=news&id=urn:contentItem:60ST-6T11-JBKR-83YD-00000-00&context=1519360.

209. (September 6, 2020 Sunday). Do you have apocalypse anxiety?. *Hindustan Times.* https://advance.lexis.com/api/document?collection=news&id=urn:contentItem:60SB-FRK1-JDKC-R3R5-00000-00&context=1519360.

210. Erin McCormick in Berkeley. (September 4, 2020 Friday). What is California's wildfire smoke doing to our health? Scientists paint a bleak picture; Research shows people, even those living hundreds of miles away, feeling the effects from wildfire smoke in the west. *The Guardian (London).* https://advance.lexis.com/api/document?collection=news&id=urn:contentItem:60RY-NXJ1-F021-63GF-00000-00&context=1519360.

211. (September 2, 2020 Wednesday). Stop preparing for a climate crisis; we're already there. *The Student Printz: University of Southern Mississippi.* https://advance.lexis.com/api/document?collection=news&id=urn:contentItem:60RT-2571-DY7P-T2X9-00000-00&context=1519360.

212. Sandy Mccarthy. (August 23, 2020 Sunday). Donald Trump declares California wildfires as major disaster, releases federal aid. *Stillman Advance: Stillman College.* https://advance.lexis.com/api/document?collection=news&id=urn:contentItem:60NK-9TX1-DY7P-T1VK-00000-00&context=1519360.

213. (August 15, 2020). In brief. *The New Zealand Herald.* https://advance.lexis.com/api/document?collection=news&id=urn:contentItem:5Y6P-9V11-JDK8-V14H-00000-00&context=1519360.

214. (August 15, 2020 Saturday). World news in brief. *The Independent - Daily Edition.* https://advance.lexis.com/api/document?collection=news&id=urn:contentItem:60KN-2B61-JCS0-D00R-00000-00&context=1519360.

215. (July 28, 2020 Tuesday). Painting Capertee trees blue for rural mental health concerns. *Lithgow Mercury.* https://advance.lexis.com/api/document?collection=news&id=urn:contentItem:60FP-M0P1-JD34-V26Y-00000-00&context=1519360.

216. (07. Juli 2020). PANORAMA. *Berliner Zeitung.* https://advance.lexis.com/api/document?collection=news&id=urn:contentItem:609B-D141-JBR8-44V6-00000-00&context=1519360.

217. (May 24, 2020 Sunday). from Corona to Karuna. *The Pioneer (India).* https://advance.lexis.com/api/document?collection=news&id=urn:contentItem:6006-MXS1-JDKC-R0BK-00000-00&context=1519360.

218. (May 7, 2020 Thursday). LETTERS. *The Age (Melbourne, Australia).* https://advance.lexis.com/api/document?collection=news&id=urn:contentItem:5YV5-SCB1-JD34-V3S4-00000-00&context=1519360.

219. (May 1, 2020 Friday). Opinion: Consequences of homelessness are devastating for communities as well as individuals. *Postmedia Breaking News.* https://advance.lexis.com/api/document?collection=news&id=urn:contentItem:5YT1-VWY1-F125-12KF-00000-00&context=1519360.

220. Dr. Will Connors, Dr. Queenie Dinh, Dr. Mary Kestler and Dr. Jan haJeK. (May 1, 2020 Friday). Pledge to curb homelessness needs urgent action. *The Vancouver Province (British Columbia).* https://advance.lexis.com/api/document?collection=news&id=urn:contentItem:5YT2-NS71-JBKR-34NB-00000-00&context=1519360.

221. (April 28, 2020 Tuesday). YOUR VIEWS. *The Gold Coast Bulletin.* https://advance.lexis.com/api/document?collection=news&id=urn:contentItem:5YS8-KP51-JD3N-52YF-00000-00&context=1519360.

222. Robert Crawford. (April 22, 2020 Wednesday). Letters to the editor. *Milton Ulladulla Times.* https://advance.lexis.com/api/document?collection=news&id=urn:contentItem:5YR1-F3J1-JD34-V1K3-00000-00&context=1519360.

223. SHANNON DEERY. (April 3, 2020 Friday). Ban crosses the line. *Herald Sun (Australia).* https://advance.lexis.com/api/document?collection=news&id=urn:contentItem:5YJY-7NR1-JD3N-50NJ-00000-00&context=1519360.

224. CHINA DAILY. (April 3, 2020 Friday). Xichang forest fire extinguished after killing 19, injuring 3; structures saved. *China Daily.* https://advance.lexis.com/api/document?collection=news&id=urn:contentItem:5YK3-5G41-F11P-X2PY-00000-00&context=1519360.

225. Olivia Laing. (March 21, 2020 Saturday). Feeling overwhelmed? How art can help in an emergency by Olivia Laing; Novels, films and paintings offer more than escapism - they provide hope, which is a vital precursor to change. *The Guardian (London).* https://advance.lexis.com/api/document?collection=news&id=urn:contentItem:5YGB-PH21-JCJY-G3NS-00000-00&context=1519360.

226. Bernhard Pötter. (Freitag 28. Februar 2020). Jenseits von Corona; Bernhard Pötter Wir retten die Welt. *taz, die tageszeitung.* https://advance.lexis.com/api/document?collection=news&id=urn:contentItem:5Y9G-B4G1-JDJ5-S2Y7-00000-00&context=1519360.

227. (February 12, 2020 Wednesday). EU crisis center monitors coronavirus outbreak, offers aid to China. *Indian Express.* https://advance.lexis.com/api/document?collection=news&id=urn:contentItem:5Y67-T8N1-JB5M-W022-00000-00&context=1519360.

228. (January 29, 2020 Wednesday). Climate Change Impacts On Health. *The Rising Nepal.* https://advance.lexis.com/api/document?collection=news&id=urn:contentItem:5Y3H-48M1-F00C-612P-00000-00&context=1519360.

229. (January 23, 2020 Thursday). Endorse agreements on drugs, mental health and violence. *CE Noticias Financieras English.* https://advance.lexis.com/api/document?collection=news&id=urn:contentItem:5Y26-27M1-DY1R-B16S-00000-00&context=1519360.

230. (Montag 20. Januar 2020). Wissen Kompakt; Scheidungen: Kinder ohne Vater sind häufiger krank ++ Waldbrandgefahr: Katastrophen bald auch im Harz? ++ Tansania: 36 Löwen sollen umgesiedelt werden. *Die Welt.* https://advance.lexis.com/api/document?collection=news&id=urn:contentItem:5Y1D-13H1-JBK9-

20NJ-00000-00&context=1519360.

231. (January 14, 2020 Tuesday). Public Health information for bushfire impacted areas. *Bombala Times.* https://advance.lexis.com/api/document?collection=news&id=urn:contentItem:5XYX-59B1-F0J6-J3JK-00000-00&context=1519360.

232. (December 02, 2019 Monday). Makgoba nominated for top science award for Aids activism. *Cape Times (South Africa).* https://advance.lexis.com/api/document?collection=news&id=urn:contentItem:5XMV-PFS1-F091-R39F-00000-00&context=1519360.

233. Newsroom. (November 18, 2019 Monday). Fire areas not out of the woods yet, but help comes to fore. *Hunter Valley & North Coast Town and Country Leader.* https://advance.lexis.com/api/document?collection=news&id=urn:contentItem:5XHR-TW31-JD34-V029-00000-00&context=1519360.

234. Dr Kathryn Woolfield Dr Kaiya Ferguson Opinion. (September 28, 2019 Saturday). Doctors stand with kids for climate action. *Sunshine Coast Daily (Queensland).* https://advance.lexis.com/api/document?collection=news&id=urn:contentItem:5XMX-BCB1-JD3N-529K-00000-00&context=1519360.

235. Sasha Letourneau, Kevin Liang And Finola Hackett. (September 17, 2019 Tuesday). Medical students take a stand on climate change. *The Vancouver Province (British Columbia).* https://advance.lexis.com/api/document?collection=news&id=urn:contentItem:5X2N-MFN1-DY2S-T1MS-00000-00&context=1519360.

236. (September 17, 2019 Tuesday). Sasha Letourneau, Kevin Liang and Finola Hackett: Medical students take a stand on climate change. *Postmedia Breaking News.* https://advance.lexis.com/api/document?collection=news&id=urn:contentItem:5X2M-G6G1-F125-100B-00000-00&context=1519360.

237. Cathy Orlando. (August 7, 2019 Wednesday). Carbon pricing good for climate change, health. *Sudbury Star.* https://advance.lexis.com/api/document?collection=news&id=urn:contentItem:5WRX-SD71-DYM8-84SW-00000-00&context=1519360.

238. (August 7, 2019 Wednesday). Sudbury letters: Carbon pricing good for climate change and health. *Sudbury Star.* https://advance.lexis.com/api/document?collection=news&id=urn:contentItem:5Y36-CXR1-JC56-J0XF-00000-00&context=1519360.

239. John Muscedere And George Heckman. (July 28, 2019 Sunday). Climate change puts the elderly at health risk. *Times Colonist (Victoria, British Columbia).* https://advance.lexis.com/api/document?collection=news&id=urn:contentItem:5WNT-M781-JBKR-9281-00000-00&context=1519360.

240. John Muscedere And George Heckman. (July 23, 2019 Tuesday). Climate and vulnerable seniors; Planning needed to protect older Canadians from climate change impact. *The Lethbridge Herald.* https://advance.lexis.com/api/document?collection=news&id=urn:contentItem:5WMR-DKG1-JC16-44C4-00000-00&context=1519360.

241. John Muscedere and George Heckman. (July 18, 2019 Thursday). Climate change places older adults at greater risk; We need to plan better to protect vulnerable seniors from the impact of climate change. *Winkler Times.* https://advance.lexis.com/api/document?collection=news&id=urn:contentItem:5WKM-XHJ1-JC58-654N-00000-00&context=1519360.

242. (July 16, 2019 Tuesday). Climate change places older adults at risk. *The Times & Transcript (New Brunswick).* https://advance.lexis.com/api/document?collection=news&id=urn:contentItem:5WK7-0671-JCT2-M44J-00000-00&context=1519360.

243. (December 20, 2018 Thursday). Pollution affects cognitive development in children. *CE Noticias Financieras English.* https://advance.lexis.com/api/document?collection=news&id=urn:contentItem:5V12-TS21-JCMP-54WX-00000-00&context=1519360.

244. Mia Rabson. (November 30, 2018 Friday). Emissions killing Canadians; Top medical journal calls on Canada to ramp up climate action, curb air pollution. *Times Colonist (Victoria, British Columbia).* https://advance.lexis.com/api/document?collection=news&id=urn:contentItem:5TVM-HBV1-DY2T-13CG-00000-00&context=1519360.

245. ALEXANDRA S. LEVINE. (November 29, 2018 Thursday). After a Wildfire, Rebuilding Life Can Be Hardest for the Oldest. *The New York Times - International Edition.* https://advance.lexis.com/api/document?collection=news&id=urn:contentItem:5TV8-4H71-JC85-N35B-00000-00&context=1519360.

246. Elizabeth Payne. (November 29, 2018 Thursday). Climate change fears spawn 'ecological grief'. *Ottawa Citizen.* https://advance.lexis.com/api/document?collection=news&id=urn:contentItem:5TVD-06J1-DY2T-8420-00000-00&context=1519360.

247. (November 29, 2018 Thursday). 'Ecological grief' among mental health effects of climate change in Canada: report. *Postmedia Breaking News.* https://advance.lexis.com/api/document?collection=news&id=urn:contentItem:5TVF-8RT1-F125-12FJ-00000-00&context=1519360.

248. (November 29, 2018 Thursday). Medical journal calls on Canada to ramp up climate action, curb air pollution. *Abbotsford News.* https://advance.lexis.com/api/document?collection=news&id=urn:contentItem:5TVN-FTR1-JDH1-C2J1-00000-00&context=1519360.

249. Elizabeth Payne. (November 29, 2018 Thursday). With 'ecological grief,'climate change goes mental. *Ottawa Sun.* https://advance.lexis.com/api/document?collection=news&id=urn:contentItem:5TVD-4S31-DYG5-Y234-00000-00&context=1519360.

250. By ALEXANDRA S. LEVINE. (November 26, 2018 Monday). Wildfires Leave Retirees Little To Rebuild On. *The New York Times.* https://advance.lexis.com/api/document?collection=news&id=urn:contentItem:5TTS-3SV1-DXY4-X2TG-00000-00&context=1519360.

251. (October 3, 2018 Wednesday). COMMENT. *Central Western Daily.* https://advance.lexis.com/api/document?collection=news&id=urn:contentItem:5TD4-2MN1-JD34-V26M-00000-00&context=1519360.

252. Madeline Smith. (August 22, 2018 Wednesday). The rise of ecoanxiety: How smoke in Alberta might affect your mental health. *The Toronto Star.* https://advance.lexis.com/api/document?collection=news&id=urn:contentItem:5T3H-1WD1-DY91-K3NR-00000-00&context=1519360.

253. Melissa Lem and Larry Barzelai. (August 9, 2018 Thursday). Wake Up And Smell The Smoke; Climate change a public health emergency, write Melissa Lem and Larry Barzelai. *The Vancouver Sun (British Columbia).* https://advance.lexis.com/api/document?collection=news&id=urn:contentItem:5T0H-NRB1-JBKR-P0D7-00000-00&context=1519360.

254. Martin Espinoza. (July 27, 2018 Friday). Rebuilding Sonoma County: Where to get and give help in fire's wake. *The Press Democrat, Santa Rosa, Calif..* https://advance.lexis.com/api/document?collection=news&id=urn:contentItem:5SWY-RWJ1-JC6P-C02H-00000-00&context=1519360.

255. COMPILED FROM NEWS SERVICES. (July 24, 2018 Tuesday). TORONTO SHOOTING KILLS 2, INJURES 13. *Pittsburgh Post-Gazette.* https://advance.lexis.com/api/document?collection=news&id=urn:contentItem:5SW4-3XF1-JC8R-353N-00000-00&context=1519360.

256. DANIELLE LE MESSURIER. (July 21, 2018 Saturday). HOME TRUTH OF ILLNESS. *The Daily Telegraph (Australia).* https://advance.lexis.com/api/document?collection=news&id=urn:contentItem:5SV9-J051-JD3N-54J4-00000-00&context=1519360.

257. Trevor Hancock. (June 24, 2018 Sunday). Carbon tax is good for our health. *Times Colonist (Victoria, British Columbia).* https://advance.lexis.com/api/document?collection=news&id=urn:contentItem:5SMR-88Y1-DY2T-137K-00000-00&context=1519360.

258. (April 26, 2018 Thursday). Lake County in 60 seconds. *Chicago Daily Herald.* https://advance.lexis.com/api/document?collection=news&id=urn:contentItem:5S66-89D1-JBRC-V2WT-00000-00&context=1519360.

259. Martin Espinoza. (April 9, 2018 Monday). 'Uncontrollable flashbacks': Mental anguish remains 6 months after fires. *The Press Democrat, Santa Rosa, Calif..* https://advance.lexis.com/api/document?collection=news&id=urn:contentItem:5S2G-YM51-JC6P-C0CN-00000-00&context=1519360.

260. Daniel Burdon and Alexandra Back. (January 23, 2018 Tuesday). A mother battles against a health system 'geared to protect itself'. *Canberra Times (Australia).* https://advance.lexis.com/api/document?collection=news&id=urn:contentItem:5RG3-YCY1-JD34-V3JN-00000-00&context=1519360.

261. (November 18, 2017 Saturday). FAMILY FORUM. *The Advertiser (Australia).* https://advance.lexis.com/api/document?collection=news&id=urn:contentItem:5R02-HCD1-F0JP-W0NC-00000-00&context=1519360.

262. (September 7, 2017 Thursday). Heavy metal toxicity and water contamination. *The Pioneer (India).* https://advance.lexis.com/api/document?collection=news&id=urn:contentItem:5PDR-6N71-JDKC-R3BP-00000-00&context=1519360.

263. (August 18, 2017 Friday). Wildfires wake us to need for climate action; Doctors know that global warming is a health issue, says Courtney Howard. *The Vancouver Sun (British Columbia).* https://advance.lexis.com/api/document?collection=news&id=urn:contentItem:5P8K-M2P1-DY2T-D232-00000-00&context=1519360.

264. Jennifer Saltman. (August 1, 2017 Tuesday). Extreme heat, smoke on the way; Air Quality: Health officials advise people in Lower Mainland to take precautions. *The Vancouver Province (British Columbia).* https://advance.lexis.com/api/document?collection=news&id=urn:contentItem:5P4Y-SN61-DY2S-T1M2-00000-00&context=1519360.

265. Jennifer Saltman. (August 1, 2017 Tuesday). Heat, air quality warnings issued for Lower Mainland. *The Vancouver Sun (British Columbia).* https://advance.lexis.com/api/document?collection=news&id=urn:contentItem:5P4Y-YD71-DY2T-D53N-00000-00&context=1519360.

266. (July 22, 2017 Saturday). So Many Challenges For Cabinet. *The Vancouver Sun (British Columbia).* https://advance.lexis.com/api/document?collection=news&id=urn:contentItem:5P2V-D261-DY2T-D29M-00000-00&context=1519360.

Bibliography

267. Deirdre Shesgreen. (June 24, 2017 Saturday). Medicaid could be deal-breaker; As opioid crisis rages, drug treatment advocates pressure senators from hard-hit states. *Dayton Daily News (Ohio).* https://advance.lexis.com/api/document?collection=news&id=urn:contentItem:5NVW-6YM1-DYGR-K4TX-00000-00&context=1519360.

268. By MICHELLE L. PRICE - Associated Press For the Deseret News. (January 2, 2017 Monday). New laws in Utah take effect in new year. *Deseret Morning News (Salt Lake City).* https://advance.lexis.com/api/document?collection=news&id=urn:contentItem:5MJG-5J61-JBRG-X30Y-00000-00&context=1519360.

269. By JEFF MARTIN Associated Press . (December 11, 2016 Sunday). Experts warn of mental health woes as wildfires ravage South. *St. Louis Post-Dispatch (Missouri).* https://advance.lexis.com/api/document?collection=news&id=urn:contentItem:5MCC-5751-DY37-34FM-00000-00&context=1519360.

270. ByJeff Martin. (December 6, 2016 Tuesday). Experts: Wildfires take toll on survivors' mental health; People have no time to prepare for flames' destruction.; WILDFIRES. *Dayton Daily News (Ohio).* https://advance.lexis.com/api/document?collection=news&id=urn:contentItem:5MB7-8BG1-JC1N-W4FC-00000-00&context=1519360.

271. Vincent Mcdermott And Dave Lazzarino. (June 25, 2016 Saturday). Sophie thanks all those who battled The Beast. *Edmonton Journal (Alberta).* https://advance.lexis.com/api/document?collection=news&id=urn:contentItem:5K37-NFV1-JBKR-152F-00000-00&context=1519360.

272. Vincent Mcdermott And Dave Lazzarino. (June 25, 2016 Saturday). Sophie thanks emergency personnel, volunteers. *The Calgary Herald (Alberta).* https://advance.lexis.com/api/document?collection=news&id=urn:contentItem:5K37-V3C1-JBKR-C0G8-00000-00&context=1519360.

273. Gordon Kent and Paige Parsons. (June 1, 2016 Wednesday). Fort McMurray evacuees brace for shock; Homecoming: Health officials fear seeing the destruction first-hand could trigger psychological trauma. *The Vancouver Province (British Columbia).* https://advance.lexis.com/api/document?collection=news&id=urn:contentItem:5JX4-HN71-DY2S-T0X5-00000-00&context=1519360.

274. (April 18, 2016 Monday). Who responds when a crisis is declared?. *The Toronto Star.* https://advance.lexis.com/api/document?collection=news&id=urn:contentItem:5JJS-1981-DY91-K2SY-00000-00&context=1519360.

275. (samedi janvier 23 2016). « Les éléments sont en place pour qu'il y ait des étincelles ». *La Dépêche du Midi.* https://advance.lexis.com/api/document?collection=news&id=urn:contentItem:5J3J-6BP1-JCJY-534P-00000-00&context=1519360.

276. Stephanie Turner. (January 18, 2016 Monday). Musician copes with Lyme disease. *Aiken Standard (South Carolina).* https://advance.lexis.com/api/document?collection=news&id=urn:contentItem:5HYG-4CT1-JC6P-C2RY-00000-00&context=1519360.

277. (August 25, 2015 Tuesday). Few new ideas in Sask. poverty plan. *The Leader-Post (Regina, Saskatchewan).* https://advance.lexis.com/api/document?collection=news&id=urn:contentItem:5GS6-R381-JBKR-41J3-00000-00&context=1519360.

278. (August 23, 2015 Sunday). FIREFIGHTING RESOURCES NEED TO BE BOLSTERED. *Spokesman Review (Spokane, WA).* https://advance.lexis.com/api/document?collection=news&id=urn:contentItem:5GRT-G2S1-JC0C-J038-

00000-00&context=1519360.

279. From staff and wire reports. (March 30, 2015 Monday). IN BRIEF. *Spokesman Review (Spokane, WA).* https://advance.lexis.com/api/document?collection=news&id=urn:contentItem:5FMN-HR81-DYFF-80NH-00000-00&context=1519360.

280. The Portola Reporter staff. (March 25, 2015). Board of Supervisors votes yes on slate of agenda items; Board of Supervisors Roundup. *Portola Reporter (California).* https://advance.lexis.com/api/document?collection=news&id=urn:contentItem:5JKD-28Y1-JDTS-G02T-00000-00&context=1519360.

281. Nora Jaara | For The Post. (November 3, 2014 Monday). Democrats heard gubernatorial running-mate Neuhardt's office goals night before election. *The Post: Ohio University.* https://advance.lexis.com/api/document?collection=news&id=urn:contentItem:5DHJ-KVH1-JBSN-33YC-00000-00&context=1519360.

282. (Samedi 2 Août 2014). *La Montagne.* https://advance.lexis.com/api/document?collection=news&id=urn:contentItem:5CTJ-7941-DXK4-B48T-00000-00&context=1519360.

283. (November 21, 2013). Climate and Mental Health - Impacts and Inequalities. *This Day (Lagos).* https://advance.lexis.com/api/document?collection=news&id=urn:contentItem:59WC-9HV1-JBJ4-20S6-00000-00&context=1519360.

284. Compiled from news services. (August 6, 2012 Monday). MARS LANDING SLATED TODAY. *Pittsburgh Post-Gazette.* https://advance.lexis.com/api/document?collection=news&id=urn:contentItem:568N-KYK1-DYRS-T3P6-00000-00&context=1519360.

285. (May 26, 2012 Saturday). Year three the danger year. *The Press (Christchurch, New Zealand).* https://advance.lexis.com/api/document?collection=news&id=urn:contentItem:55R4-XXK1-DYTR-J335-00000-00&context=1519360.

286. CHRIS JOHNSTON. (May 19, 2012 Saturday). Ashes to ashes: forgotten fire town mourns again. *The Age (Melbourne, Australia).* https://advance.lexis.com/api/document?collection=news&id=urn:contentItem:55NN-1G21-F0J6-J4D2-00000-00&context=1519360.

287. Chris Johnston. (May 19, 2012 Saturday). Kinglake 'stuck in a perpetual crisis'. *Canberra Times (Australia).* https://advance.lexis.com/api/document?collection=news&id=urn:contentItem:55NN-39B1-DYTG-20HM-00000-00&context=1519360.

288. The New Mexican. (February 8, 2012 Wednesday). LEGISLATIVE ROUNDUP. *The Santa Fe New Mexican (New Mexico).* https://advance.lexis.com/api/document?collection=news&id=urn:contentItem:55C1-CSS1-DY7H-R07W-00000-00&context=1519360.

289. JUDY SIEGEL. (January 12, 2012 Thursday). Orlev slams government for failing to reform mental-health services. National Council of the Child head Dr. Yitzhak Kadman decries 'shocking' pediatric psychiatric treatment, 'collapsing' facilities. *Jerusalem Post.* https://advance.lexis.com/api/document?collection=news&id=urn:contentItem:54S1-P201-JDKD-P22K-00000-00&context=1519360.

290. (December 26, 2011 Monday). Global warming, natural disasters, climate change and health. *Daily News (Sri Lanka).* https://advance.lexis.com/api/document?collection=news&id=urn:contentItem:54JR-RHB1-JDKC-R3XK-00000-00&context=1519360.

291. JUDY SIEGEL-ITZKOVICH. (December 18, 2011 Sunday). For rescuers, effects of the Carmel Forest fire linger on. *Jerusalem Post.*

https://advance.lexis.com/api/document?collection=news&id=urn:contentItem:54J3-5S01-F12G-D15V-00000-00&context=1519360.

292. ERIC AMSTER . (April 7, 2011 Thursday). And those that lived? . *Jerusalem Post.* https://advance.lexis.com/api/document?collection=news&id=urn:contentItem:52KX-B2F1-F12G-D2NK-00000-00&context=1519360.

293. (January 19, 2011). Water-borne disease seems almost certain. *Capricorn Coast Mirror (Queensland).* https://advance.lexis.com/api/document?collection=news&id=urn:contentItem:5200-T2F1-F149-S2JS-00000-00&context=1519360.

294. Wolfgang Merkel. (Dienstag 10. August 2010). Das giftige Gas von Moskau; Russlands Waldbrände: Kohlenmonoxid bedroht sogar ungeborene Kinder. *Welt kompakt.* https://advance.lexis.com/api/document?collection=news&id=urn:contentItem:804N-0R71-2SMY-M1B4-00000-00&context=1519360.

295. Wolfgang W. Merkel. (Dienstag 10. August 2010). Kohlenmonoxid raubt dem Körper Sauerstoff; Durch die russischen Torf- und Waldbrände sind vor allem Herz-Kreislauf-Kranke, körperlich hart Arbeitende und Föten bedroht. *Die Welt.* https://advance.lexis.com/api/document?collection=news&id=urn:contentItem:804N-C660-Y8XK-M17Y-00000-00&context=1519360.

296. JIM EFSTATHIOU NEW YORK. (November 22, 2009 Sunday). Illness rises with heat; Climate change increases sickness. *The Sydney Morning Herald.* https://advance.lexis.com/api/document?collection=news&id=urn:contentItem:7X51-GDF1-2RR2-D0P1-00000-00&context=1519360.

297. NEW MEXICAN WIRE SERVICES. (October 5, 2009 Monday). IN BRIEF. *The Santa Fe New Mexican (New Mexico).* https://advance.lexis.com/api/document?collection=news&id=urn:contentItem:7WSV-SF00-Y93S-K0WF-00000-00&context=1519360.

298. (April 7, 2009 Tuesday). National Journal. *The Telegraph-Journal (New Brunswick).* https://advance.lexis.com/api/document?collection=news&id=urn:contentItem:7VD5-S4B0-YB4C-70X7-00000-00&context=1519360.

299. (November 19, 2008 Wednesday). METROWorld. *Metro (UK).* https://advance.lexis.com/api/document?collection=news&id=urn:contentItem:4TYK-5450-TYBR-71K7-00000-00&context=1519360.

300. (November 4, 2007 Sunday). Needle exchange good for users, not others. *Times Colonist (Victoria, British Columbia).* https://advance.lexis.com/api/document?collection=news&id=urn:contentItem:4R28-YV30-TWD3-K0GF-00000-00&context=1519360.

301. RAPHAËLLE THOMAS. (26 juillet 2007). Vague de chaleur dévastatrice en Europe du Sud. *Le Figaro.* https://advance.lexis.com/api/document?collection=news&id=urn:contentItem:4P8P-3R70-TWKR-42BK-00000-00&context=1519360.

302. THE NEW MEXICAN. (June 28, 2007 Thursday). IN BRIEF. *The Santa Fe New Mexican (New Mexico).* https://advance.lexis.com/api/document?collection=news&id=urn:contentItem:4P3B-6R50-TWJV-92KJ-00000-00&context=1519360.

303. Justin Nobel. (April 6, 2007 Friday). Eco-anxiety epidemic; Doomsday fears worry some people sick. *Edmonton Journal (Alberta).* https://advance.lexis.com/api/document?collection=news&id=urn:contentItem:4NF2-1YW0-TWD3-91XY-00000-00&context=1519360.

304. FROM NEWS SERVICES. (February 8, 2006 Wednesday). DIGEST. *St. Louis Post-Dispatch (Missouri).* https://advance.lexis.com/api/document?collection=news&id=urn:contentItem:4J74-KJ10-TWDH-N2YG-00000-00&context=1519360.

305. Peter O'Neil. (January 6, 2006 Friday). Liberals bring out goody bag for B.C.: Party to unveil spending promises today in Vancouver. *The Vancouver Sun (British Columbia).* https://advance.lexis.com/api/document?collection=news&id=urn:contentItem:4J01-M9R0-TWD4-033B-00000-00&context=1519360.

306. Peter O'Neil. (January 6, 2006 Friday). Liberals roll out B.C. spending plans: West Coast MPs, candidates vow to deliver major projects. *The Vancouver Sun (British Columbia).* https://advance.lexis.com/api/document?collection=news&id=urn:contentItem:4J01-M9R0-TWD4-0338-00000-00&context=1519360.

307. Peter O'Neil. (January 6, 2006 Friday). Liberals target B.C. with wish list agenda: Natural disaster centre proposed for prone West Coast. *National Post (f/k/a The Financial Post) (Canada).* https://advance.lexis.com/api/document?collection=news&id=urn:contentItem:4J01-9W40-TWD3-S2GV-00000-00&context=1519360.

308. Peter O'Neil. (January 6, 2006 Friday). Liberals want 'to flatter' B.C. with natural disaster centre: Wish list to be unveiled today. *National Post (f/k/a The Financial Post) (Canada).* https://advance.lexis.com/api/document?collection=news&id=urn:contentItem:4J01-9W40-TWD3-S2GD-00000-00&context=1519360.

309. (October 7, 2005). Guardian Weekly: Outlook: Field Notes. *Guardian Weekly.* https://advance.lexis.com/api/document?collection=news&id=urn:contentItem:4HB3-X8W0-TX1F-4389-00000-00&context=1519360.

310. (September 1, 2003 Monday). Saluting those who toil. *The Toronto Star.* https://advance.lexis.com/api/document?collection=news&id=urn:contentItem:49F2-JGG0-006F-02W9-00000-00&context=1519360.

311. (November 29, 2002, Friday). *Manly Daily (Australia).* https://advance.lexis.com/api/document?collection=news&id=urn:contentItem:47B2-WW10-015H-J4GK-00000-00&context=1519360.

312. (June 14, 2000, Wednesday). Diary. *South-West News.* https://advance.lexis.com/api/document?collection=news&id=urn:contentItem:44JN-7X10-0198-32MD-00000-00&context=1519360.

313. (MAY 1, 2000 Monday SF EDITION). NEWS IN BRIEF. *The Philadelphia Inquirer.* https://advance.lexis.com/api/document?collection=news&id=urn:contentItem:452N-TF00-0190-X114-00000-00&context=1519360.

314. THE GAZETTE. (October 11, 1997, Saturday, FINAL EDITION). EarthWeek. *The Gazette (Montreal, Quebec).* https://advance.lexis.com/api/document?collection=news&id=urn:contentItem:3SR8-SFJ0-002G-H0HH-00000-00&context=1519360.

315. (September 27, 1997 Saturday Final Edition). Smog may pose deadly hazard. *The Record (Kitchener-Waterloo, Ontario).* https://advance.lexis.com/api/document?collection=news&id=urn:contentItem:46W8-ND00-01GV-M041-00000-00&context=1519360.

316. (August 12, 1993 Thursday Final Edition). STILL OPEN FOR BUSINESS. *Hamilton Spectator (Ontario, Canada).* https://advance.lexis.com/api/document?collection=news&id=urn:contentItem:473C-35C0-01GV-K4MS-00000-00&context=1519360.

317. JUDY SIEGEL. (Thursday, January 12, 2011). Orlev slams government for failing to reform mental-health services. National Council of the Child head Dr. Yitzhak Kadman decries 'shocking' pediatric psychiatric treatment, 'collapsing' facilities. *Jerusalem Post.* https://advance.lexis.com/api/document?collection=news&id=urn:contentItem:54RV-50S1-F12G-D18S-00000-00&context=1519360.

End of Document
